# USP43‐mediated deubiquitination of SLC7A11 protects against LPS‐induced acute lung injury by inhibiting ferroptosis

**DOI:** 10.1002/ctm2.70718

**Published:** 2026-06-30

**Authors:** Li Zhang, Jutong He, Ming Xu, Bamawa Mwengendi Joël, Guiomar Correia, Xuefeng Zhou, Xinyi Li, Hexiao Tang

**Affiliations:** ^1^ Department of Thoracic Surgery Zhongnan Hospital of Wuhan University Wuhan China; ^2^ Center for Animal Experiment Wuhan University School of Medicine Wuhan China; ^3^ Department of General Practice Mboluoni Medical Center Kisangani University Kisangani Democratic Republic of Congo; ^4^ Department of Cardiovascular and Thoracic surgery Cardinal Cardiopulmonary Diseases Hospital Luanda Angola; ^5^ Department of Anaesthesiology Zhongnan Hospital of Wuhan University Wuhan China

**Keywords:** acute lung injury, ferroptosis, SLC7A11, ubiquitination, USP43

## Abstract

**Background:**

Acute lung injury (ALI) is a common inflammatory pulmonary disorder, with increasing evidence implicating ferroptosis as a critical type of cell death in its pathogenesis. Ubiquitin‐specific protease 43 (USP43) is an important deubiquitinating enzyme that plays a significant role in both inflammation and ferroptosis regulation. In this study, we mainly analysed whether USP43 could participate in the process of ALI by regulating the ferroptosis process, and clarified its molecular mechanism.

**Methods:**

To investigate the functional role of USP43 in ALI, *Usp43* knockout mice, *USP43* knockdown and overexpressed human bronchial epithelial BEAS‐2B cells and mouse alveolar type II epithelial MLE12 cells were treated with lipopolysaccharide (LPS) in vivo and in vitro. Subsequently, ferroptosis inhibitor and activator, Ferrostatin‐1 and Erastin, were taken to explore the effect of ferroptosis on the regulation function of USP43 in ALI. Finally, co‐immunoprecipitation (Co‐IP), ubiquitination and rescue assays were conducted to determine the regulation mechanism of USP43.

**Results:**

The expression of USP43 was up‐regulated during ALI. *Usp43*‐KO mice exhibited aggravated lung injury, inflammation and ferroptosis. Consistently, *USP43* knockdown exacerbated LPS‐stimulated cellular damage, inflammation and ferroptosis, while its overexpression exerted the opposite effect in vitro. Furthermore, the regulation effects of USP43 on ALI mainly depended on ferroptosis by administering a ferroptosis inducer and inhibitor, respectively. Mechanistically, USP43 was found to inhibit K48‐linked polyubiquitination of solute carrier family 7 member 11 (SLC7A11), thereby stabilising SLC7A11. Overexpression of SLC7A11 rescued the ferroptosis, cellular and lung tissue injury and inflammation aggravated by USP43 knockdown or deficiency.

**Conclusion:**

USP43 prevents the progression of ALI by mediating K48‐linked deubiquitination of SLC7A11, thereby inhibiting ferroptosis. Targeting USP43 may represent a potential therapeutic strategy for ALI treatment by enhancing SLC7A11 stability and inhibiting ferroptosis.

**Key points:**

- USP43 has been proven to be upregulated during LPS‐induced ALI.
- This study is the first to demonstrate that USP43 can inhibit the progression of LPS‐induced ALI by suppressing ferroptosis.
- This study proved that USP43 can interects with SLC7A11 and removes the K48‐linked ubiquitinaiton of SLC7A11, thereby enhances the stability of it and subsequently inhibiting ferroptosis during the LPS-induced ALI process.
- The regulation of USP43 on LPS‐induced ALI mainly depends on SLC7A11, and USP43 is expected to become a new target for the treatment of ALI/ADRS.

## Graphical abstract

1. Ubiquitin‐specific protease 43 (USP43) in up‐regulated during lipopolysaccharide (LPS)‐induced acute lung injury (ALI).
2. USP43 interacts and inhibits the degradation of solute carrier family 7 member 11 (SLC7A11) by removing the K48‐linked ubiquitination of it, thereby suppressing ferroptosis.
3. USP43 inhibits the progression of ALI by suppressing ferroptosis.

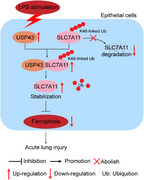

## 1 INTRODUCTION

Acute lung injury (ALI) is an inflammatory pulmonary disorder that can be triggered by various factors, including severe infections (e.g., sepsis), trauma, aspiration and pancreatitis.^1^ Among these, infection represents the most prevalent cause, with lipopolysaccharide (LPS) serving as a critical inflammatory trigger.^2^ When progressing to its severe form, ALI develops into acute respiratory distress syndrome (ARDS), a life‐threatening condition frequently encountered in critical care medicine. Despite advances in mechanical ventilation and intensive care management, ALI/ARDS continues to carry a high mortality rate of nearly 40%.^3^, ^4^ Therefore, clarifying the new mechanisms underlying the pathogenesis and progression of ALI/ARDS is of great clinical significance for the development of new therapeutic approaches and drugs for ALI/ARDS.

Ferroptosis, a novel form of cell death that is distinct from apoptosis, necrosis and autophagy, is characterised by the excessive accumulation of lipid peroxidation resulting from the disruption of intracellular metabolic pathways.^5^ Ferroptosis contributes greatly to the occurrence and development of ALI. Recent studies have shown that METTL3 can regulate ferroptosis in sepsis‐associated ALI by mediating the m6A modification of ACSL4.^6^ Obacunone can activate Nrf2 by inhibiting its ubiquitination degradation, thereby inhibiting ferroptosis and alleviating LPS‐induced ALI.^7^ Additionally, p53 suppression has been reported to activate the solute carrier family 7 member 11 (SLC7A11) pathway, thereby reducing ferroptosis in lung epithelial cells and inhibiting ALI induced by LPS.^8^, ^9^ Therefore, further clarification of the mechanism of ferroptosis in ALI is expected to provide a new theoretical basis for the development of drugs for treating ALI by targeting ferroptosis regulation.

Ubiquitin‐specific protease 43 (USP43) belongs to the deubiquitinase (DUB) family, which mediates the cleavage of ubiquitin chains from target proteins and then regulating protein stability and signalling pathways. Emerging studies have gradually unveiled the multifaceted functions of USP43 in human diseases, particularly in cancer biology. Accumulating evidence demonstrates that USP43 generally exhibits tumour‐promoting effects through stabilising oncoproteins or modulating key signalling pathways. For instance, USP43 promotes bladder cancer progression by removing K48‐linked ubiquitin chains from the oncogene c‐Myc, thereby stabilising it.^10^ In ovarian cancer, USP43 triggers the Wnt/β‐catenin pathway by deubiquitinating HDAC2, consequently reducing cisplatin sensitivity.^11^ Additionally, in cervical cancer, USP43 facilitates tumour progression by stabilising TAZ expression and activating the Hippo/TAZ pathway.^12^ Beyond its oncogenic roles, USP43 has also been implicated in metabolic and inflammatory processes. It alleviates diabetic kidney disease through HSPA8 deubiquitination.^13^ Upon viral infection, USP43 regulates antiviral innate immune responses through its deubiquitination of the E3 ubiquitin ligase RNF2, which in turn facilitates TBK1 ubiquitination and proteasomal degradation.^14^ In addition, the latest research also indicates that USP43 can stabilise FASN through deubiquitination, thereby activating the expression of SLC7A11 and inhibiting the process of ferroptosis in ovarian cancer.^15^ Nevertheless, the potential involvement of USP43 in ALI remains completely unexplored.

In this study, we hypothesised that USP43 may participate in the progression of ALI by regulating ferroptosis. To verify this hypothesis, *Usp43* knockout (KO) mice, *USP43* knockdown and overexpressed BEAS‐2B and MLE12 cells were used to established ALI model by stimulating with LPS. By evaluating the lung tissue damage, alveolar permeability, cell damage and the intensity of inflammatory response activation, the influence of USP43 on ALI was analysed. The Fe^2+^ and lipid peroxidation biochemical tests and the expressions of SLC7A11 and GPX4 detection were used to verify the effects of USP43 on ferroptosis. The molecular mechanism by which USP43 regulates ALI was explored through molecular biology experiments including co‐immunoprecipitation (Co‐IP), glutathione S‐transferase (GST)‐pulldown, mapping and ubiquitination studies. Finally, functional rescue experiments were conducted to determine whether the regulation of USP43 on ALI mainly depends on its regulation of ferroptosis both in vitro and in vivo. The aim of this investigation was to verify the role of USP43 on ALI and elucidate the molecular mechanism by which USP43 modulates ALI progression.

## 2 METHODS

### 2.1 Cell culture and ALI model

Human embryonic kidney cells (HEK293T), human bronchial epithelial BEAS‐2B cells and mouse alveolar type II epithelial MLE12 cells were obtained from American Type Culture Collection and cultured in Dulbecco's Modified Eagle Medium medium (Procell, PM150210) supplemented with 10% foetal bovine serum (Newzerum, FBS‐UE500), 1% penicillin–streptomycin solution (Procell, PB180120). Cells were maintained at 37°C in a humidified incubator with 5% CO_2_. All cell lines were confirmed to be free of mycoplasma. BEAS‐2B and MLE12 cells were treated with 1 µg/mL LPS for 24 h to establish an in vitro model of lung injury. The ferroptosis inhibitor, Ferrostatin‐1 (Fer‐1, 2 µM) or inducer Erastin (20 µM), was added to culture 2 h or 24 h before LPS exposure.

### 2.2 Antibodies and reagents

Antibodies: Anti‐SLC7A11 (ABclonal, A2413), Anti‐USP43 (HUABIO, ER65281), Anti‐GPX4 (ABclonal, A25009), Anti‐ACTIN (HUABIO, EM21002), Anti‐Flag (MBL, M185‐3L), Anti‐Myc (ABclonal, AE070), Anti‐HA (ABclonal, AE105).

Reagents: LPS (Sigma‐Aldrich, L2630), MG132 (MCE, HY‐13259), Chloroquine (CQ; MCE, HY‐17589A), Cycloheximide (CHX; GLP, GC17198), DMSO (Beyotime, ST038), Ferrostatin‐1 (Fer‐1, MCE, HY‐100579), Erastin (MCE, HY‐15763).

### 2.3 Bioinformatics analysis

The expression level of *Usp43* was analysed using dataset GSE263867 (GEO Accession viewer). The dataset of Control group (five samples) and pulmonary ALI group (five samples, intratracheal injection of LPS) were download for subsequent analysis. Raw RNA‐seq reads were normalised using the transcripts per million (TPM) method. The statistical significance of inter‐group differences in gene expression was determined using a *t*‐test. Data visualisation was performed using boxplots.

### 2.4 Generation of stable USP43‐modified BEAS‐2B and MLE12 cells

To overexpress USP43 and SLC7A11 in BEAS‐2B cells, the human *USP43* and *SLC7A11* coding DNA sequences (CDS) were amplified and subcloned into the pLVX vector using the ClonExpress II One Step Cloning Kit (Vazyme, C112‐02). The USP43 overexpression recombinant plasmid (pLVX‐HA‐*USP43*) and SLC7A11 overexpression recombinant plasmid (pLVX‐Flag‐*SLC7A11*) were obtained. To knock down USP43 and SLC7A11 in BEAS‐2B cells, a short hairpin RNA (shRNA) sequence targeting the human *USP43* or *SLC7A11* gene was designed and synthesised, and then inserted into the pLKO.1 vector to obtain the USP43 knockdown or SLC7A11 knockdown recombinant plasmid (pLKO.1‐sh*USP43*, pLKO.1‐sh*SLC7A11*). These obtained plasmid was co‐transfected with packaging plasmids (psPAX2/pMD2.G) using polyethylenimine (Polysciences, 24765) into HEK293T cells to produce virus. The obtained viruses were employed to infect BEAS‐2B cells with 5 µg/mL Polybrene (Sigma‐Aldrich, TR‐1003) added. The positive cells were selected with 2 µg/mL puromycin for 7 days, and knockdown or overexpression efficiency was validated by Western blot. To overexpress or knockdown Usp43 in MLE12 cells, CDS region amplification primers or shRNA sequences were designed for the mouse *Usp43* gene. The remaining methods are the same as those used in BEAS‐2B. Control cells received empty vector (pLVX) or non‐targeting shRNA. The primer sequences were listed in Supporting Information Table 1.

### 2.5 Animals and acute lung injury model

Eight‐ to ten‐week‐old C57BL/6J wild‐type (WT) mice, *Usp43*‐knockout (KO) mice and its littermates weighing 25–30 g were used as the experimental subjects. To establish ALI model, the mice were anesthetised with sodium pentobarbital (50 mg/kg, injected intraperitoneally), followed by intratracheal administration of 50 µL LPS (10 mg/kg) using a micro‐spray aerosol injector. The control group was treated with the same volume of phosphate buffered saline (PBS). Twenty‐four hours after LPS stimulation, the mice were euthanised and sacrificed, the lung tissues and bronchoalveolar lavage fluid (BALF) were collected for subsequent analysis.

The animal experiment was approved by the Animal Ethics Committee of Zhongnan Hospital of Wuhan University (approval number ZN2025174). Considering animal ethics, mice presenting severe dyspnoea and poor physical activity during LPS challenge would be excluded.

To evaluate the regulatory effect of USP43 on ALI, 60 *Usp43*‐KO mice and 60 WT littermates were selected to establish an in vivo ALI model. The mice were randomly divided into four groups (*n* = 30 per group): WT PBS group, *Usp43*‐KO PBS group, WT LPS group, *Usp43*‐KO LPS group. During the LPS stimulation process, one mouse in the WT LPS group and two mice in the KO LPS group died. Apart from these, no additional animals were excluded.

To overexpress Slc7a11 in mice lung tissue, the adeno‐associated viral (AAV)‐6 vectors mediated *Slc7a11* overexpression with Sftpc promoter and its control virus (AAV6‐Ctrl) were constructed by Design Bio (Wuhan). 60 *Usp43*‐KO mice and 60 WT mice were randomly divided into the following groups (*n* = 30 per group): WT + AAV6‐Ctrl LPS group, *Usp43*‐KO + AAV6‐Ctrl LPS group, WT + AAV6‐*Slc7a11* LPS group, *Usp43*‐KO + AAV6‐*Slc7a11* LPS group. The mice were anesthetised with pentobarbital sodium and intratracheally injected corresponding virus (5 × 10^12^ vg/mL, 50 µL/mice). Four weeks later, the LPS was administrated intratracheally according to the description above.

Twenty‐four hours after LPS stimulation, animals in each group were randomly allocated to different detection endpoints according to previous report^16^, ^17^, ^18^: eight mice per group were used for BALF collection; eight mice per group were used for lung wet/dry weight measurement; six mice per group were used for Evans Blue staining and pulmonary vascular permeability detection; the remaining mice (six to eight mice per group) were sacrificed for histological staining and molecular biological evaluation.

### 2.6 Pathological analysis

The mice were anesthetised and sacrificed. After perfusing the lung with normal saline to remove the residual blood, the left lung was fixed in 10% formalin, and the right lung was rapidly frozen in liquid nitrogen. After dehydration and paraffin embedding, the fixed lung tissues were cut into 5 µm thick sections. The sections were dewaxed in xylene and rehydrated through a graded alcohol series.

For haematoxylin and eosin (H&E) staining, the sections were stained with haematoxylin for 5 min. After rinsing in tap water and differentiation in 1% acid alcohol, sections were blued in Scott's solution, counterstained with eosin for 2 min, dehydrated, cleared in xylene, and mounted. Lung injury was scored by an observer blinded to the experimental groups. The scoring criteria included four parameters: alveolar wall thickening, inflammatory cell infiltration, haemorrhage and pulmonary oedema. Each parameter was graded on a scale of 0–4 (0 = none, 1 = mild [≤25% lung involvement], 2 = moderate [25%–50% lung involvement], 3 = severe [50%–75% lung involvement], 4 = very severe [75%–nearly total lung involvement]). Six high‐power fields (HPFs) were randomly captured for each sample. The lung injury score for each field was the sum of the scores of all indicators, and the average value was taken as the injury score for each mouse.^19^, ^20^

For immunofluorescence staining, following antigen retrieval and blocking treatment, the sections were incubated with anti‐CD11b primary antibody (Boster, BM3925) overnight at 4°C. After washing with PBS for three times, the sections were incubated with fluorophore‐conjugated secondary antibody (Servicebio, GB28301). Nuclei were then counterstained with DAPI (Beyotime, P0131), and slides were mounted with antifade medium.

For immunohistochemical staining, lung tissue sections were subjected to antigen retrieval using sodium citrate solution (Solarbio, Beijing, C1032) under high temperature and pressure conditions. After retrieval, goat serum (Boster, AR0009) was added and incubated at 37°C for 30 min to block nonspecific binding. Then, the blocking solution was discarded, and primary antibodies (anti‐Ly6g [Servicebio, GB11229], anti‐F4/80 [Servicebio, GB11027]) were diluted and incubated overnight at 4°C. Following incubation, the sections were then washed three times again with PBS. Then, a ready‐to‐use immunohistochemistry polyclonal secondary antibody (Servicebio, G1302) was applied and incubated at 37°C for 1 h. After washing three more times with PBS, diaminobenzidine substrate solution (ZSGB‐Bio, ZLI‐9018) was used for colour development. Nuclei were counterstained with haematoxylin, and coverslips were mounted with neutral gum (Biosharp, BL704A).

For positive‐staining cells quantification, a researcher who was not familiar with the group carried out the process using Image‐Pro Plus software (version 2.2). At least six HPFs from each mouse's lung section were selected for quantitative analysis. The results are presented as the average number of positively stained cells per HPFs.

### 2.7 Lung wet weight to dry weight ratio detection

Twenty‐four hours after LPS stimulation, eight mice in each group were anesthetised and sacrificed. The lung tissues were collected and weighed. To obtain the dry weight, the lung tissues were placed in a 70°C oven until the weight no longer changes. Then, the wet weight to dry weight (W/D) ratio was calculated.

### 2.8 Collection and testing of bronchoalveolar lavage fluid

Twenty‐four hours after LPS stimulation, eight mice in each group were anesthetised and sacrificed. The exposed trachea was cannulated with a 16‐gauge (16 G) needle. Subsequently, the lungs were lavaged three times with 1 mL of ice‐cold normal saline. The collected BALF was centrifuged at 800 × *g* at 4°C for 10 min. The supernatant was collected and the protein content in BALF was detected using the bicinchoninic acid kit (Abbkine, KTD3001). The precipitated cells were lysed with red blood cell lysis solution, and the total cell count was performed using the blood cell counter (Auto 1000, Nexcelom).

### 2.9 Assessment of pulmonary capillary leakage

Thirty minutes prior to the end of stimulation, six mice per group were anesthetised and intravenously injected with Evans Blue dye (40 mg/kg) via the tail vein. After 30 min of circulation, the mice were anesthetised with sodium pentobarbital (50 mg/kg, i.p.) and sacrificed. After perfusion with normal saline through the right ventricle to clear intravascular dye, the lungs were then carefully excised, gently inflated with air to fully expand the lobes for photographic documentation. The lung tissues were weighed for subsequent Evans Blue quantification in lung tissues.

### 2.10 Determination of related indicators of ferroptosis

The BODIPY 581/591 C11 kit (Servicebio, G1733) was used to stain the lipid peroxidation in the cells, and the observation was conducted using a fluorescence microscope. The levels of Fe^2+^, malondialdehyde (MDA), glutathione (GSH), oxidised glutathione (GSSG) and NADPH/NADP^+^ in lung tissues and cells were quantified using corresponding assay kits (Fe^2+^, Beyotime, S1068M; MDA, Beyotime, S0131; GSH and GSSG, Beyotime, S0053; NADPH/NADP^+^, Beyotime, S0179) according to the manufacturers’ protocols.

### 2.11 Western blot

Cells were lysed in radio immunoprecipitation assay (RIPA) lysis buffer (65 mM Tris–HCl [pH 7.5], 150 mM NaCl, 1 mM EDTA, 1% NP‐40 [Solarbio, N8030], .5% sodium deoxycholate, .1% SDS) containing protease inhibitor cocktail (Roche, 4693132001) on ice for 30 min. Tissues were minced and homogenised in RIPA buffer using a tissue grinder. The supernatants were collected after centrifuged at 12 000 rpm for 15 min at 4°C. The obtained protein samples were separated by 10% sodium dodecyl sulfate (SDS)‐PAGE. Subsequently, it was transferred to polyvinylidene fluoride membranes (Millipore, IPVH00010). After blocking with 5% non‐fat milk, the membranes were incubated with corresponding primary antibodies overnight at 4°C. After washing with TBST for 3 times, the membranes were then exposed to horseradish peroxidase‐conjugated secondary antibodies (1:50 000, Jackson, 111‐035‐003, 115‐035‐003) for 1 h at room temperature. Band visualisation was performed using an enhanced chemiluminescence detection kit (Epizyme, SQ201L) and protein quantitative analysis was performed with ImageJ software.

### 2.12 RNA isolation and reverse transcription‐polymerase chain reaction (RT‐PCR) analysis

Total RNA was extracted using TRIzol reagent (TIANGEN, 4992730) and reverse transcribed into cDNA using HiScript III RT SuperMix (Tsingke, TSK314M). Polymerase chain reaction (PCR) was performed using ChamQ SYBR qPCR Master Mix (Vazyme, Q311‐02) following cycling conditions: 95°C for 30 s, then 40 cycles of 95°C for 10 s and 60°C for 30 s. Gene expression levels were normalised to *ACTB* and calculated using the 2^−ΔΔCt^ method. The primer sequences were listed in Supporting Information Table 2.

### 2.13 Cell activity assay and lactate dehydrogenase detection

Ninety‐six‐well plates were seeded with cells at a density of 5 × 10^3^ cells per well and let them adhere overnight. Following treatment with LPS for 24 h, 10 µL of CCK‐8 reagent (Beyotime, C0039) was added to each well, and the plates were incubated at 37°C for 2 h. Absorbance was then detected at 450 nm using a microplate reader, and cell activity was calculated. The lactate dehydrogenase (LDH) content in the culture supernatant was assessed using a commercial LDH cytotoxicity detection kit (Beyotime, C0019S) according to the manufacturer's instructions.

### 2.14 Detection of inflammatory factors

The levels of inflammatory factors in BALF and cell culture supernatants were quantified by enzyme‐linked immunosorbent assay (ELISA) kits from Abclonal.

### 2.15 Plasmids construct

The full‐length CDS region of human *USP43*, *SLC7A11* and *GPX4* genes were cloned into the pHAGE‐HA or pcDNA5‐Flag‐Cherry vector separately. The plasmids encoding truncated USP43, SLC7A11 and a dominant‐negative USP43 (USP43(C110S)) were constructed via cloning their respective coding regions into the pHAGE‐HA or pcDNA5‐Flag‐Cherry vector. The primers used for plasmid construction are listed in Supporting Information Table 3.

### 2.16 Co‐immunoprecipitation assay

Cells were co‐transfected with plasmids encoding potential interacting proteins for interaction validation. After 48 h, cells were lysed in mixed buffer (20 mmol/L Tris–HCl (pH 7.4), 150 mM NaCl, 1 mM EDTA, 1% NP‐40 and protease inhibitor cocktail). After centrifugation, an anti‐HA or anti‐Flag antibody of HEK293T cells was added to the supernatants to incubate overnight at 4°C, followed by Protein A/G bead capture for 5 h. Then the bound proteins were eluted from the beads with SDS loading buffer. Input and immunoprecipitated samples were analysed by Western blot.

### 2.17 GST pull‐down assay

GST‐HA‐USP43 or GST‐HA‐SLC7A11 plasmids were transfected into HEK293T cells. After 48 h, the cells expressing GST‐tagged proteins were lysed. The lysates were mixed with glutathione–sepharose beads to purify the GST‐fusion proteins. The lysates of HEK293T cells overexpressing Flag‐SLC7A11 or Flag‐USP43 were incubated with purified beads overnight. Bound proteins were eluted with SDS loading buffer and examined by Western blot.

### 2.18 Ubiquitination assay

To assess the ubiquitination level of SLC7A11, cells co‐transfected with Flag‐SLC7A11 and Myc‐Ub plasmids were treated with 10 µM MG132 for the final 6–8 h of the 48‐h post‐transfection period before harvest. Cells were lysed in 1% SDS buffer (50 mM Tris–HCl, 150 mM NaCl) and boiled at 95°C for 5–10 min, followed by 10‐fold dilution with NP‐40 buffer to reduce SDS concentration to 0.1% for subsequent IP. Lysates were subjected to immunoprecipitation with anti‐Flag antibody overnight at 4°C, followed by Protein A/G bead capture. After stringent washing with a high‐salt buffer, the bound proteins were eluted in SDS loading buffer and analysed by Western blot.

### 2.19 Statistical analysis

GraphPad Prism 8 was used for data visualisation. All data in the figures are presented as mean ± standard deviation (SD). The statistical analysis was performed using IBM SPSS Statistics 21 software. During our statistical analysis, the Shapiro–Wilk test was used to perform the normal distribution analysis. When the data followed a normal distribution, a two‐tailed Student's *t*‐test was used to compare the significance between two groups. A one‐way ANOVA analysis was used to compare the differences between each pair of the three or more groups of data. When conducting the one‐way ANOVA analysis, the Bonferroni post‐hoc test was used to assess the homogeneity of the data variance, while the Tamhane's T2 (M) post‐hoc test was employed to evaluate the heterogeneity of the data variance. The data that deviated from the normal distribution were subjected to analysis using nonparametric statistical tests. *p* < .05 was considered to indicate statistical significance.

## 3 RESULTS

### 3.1 USP43 is up‐regulated in LPS‐induced acute lung injury

**FIGURE 1:**
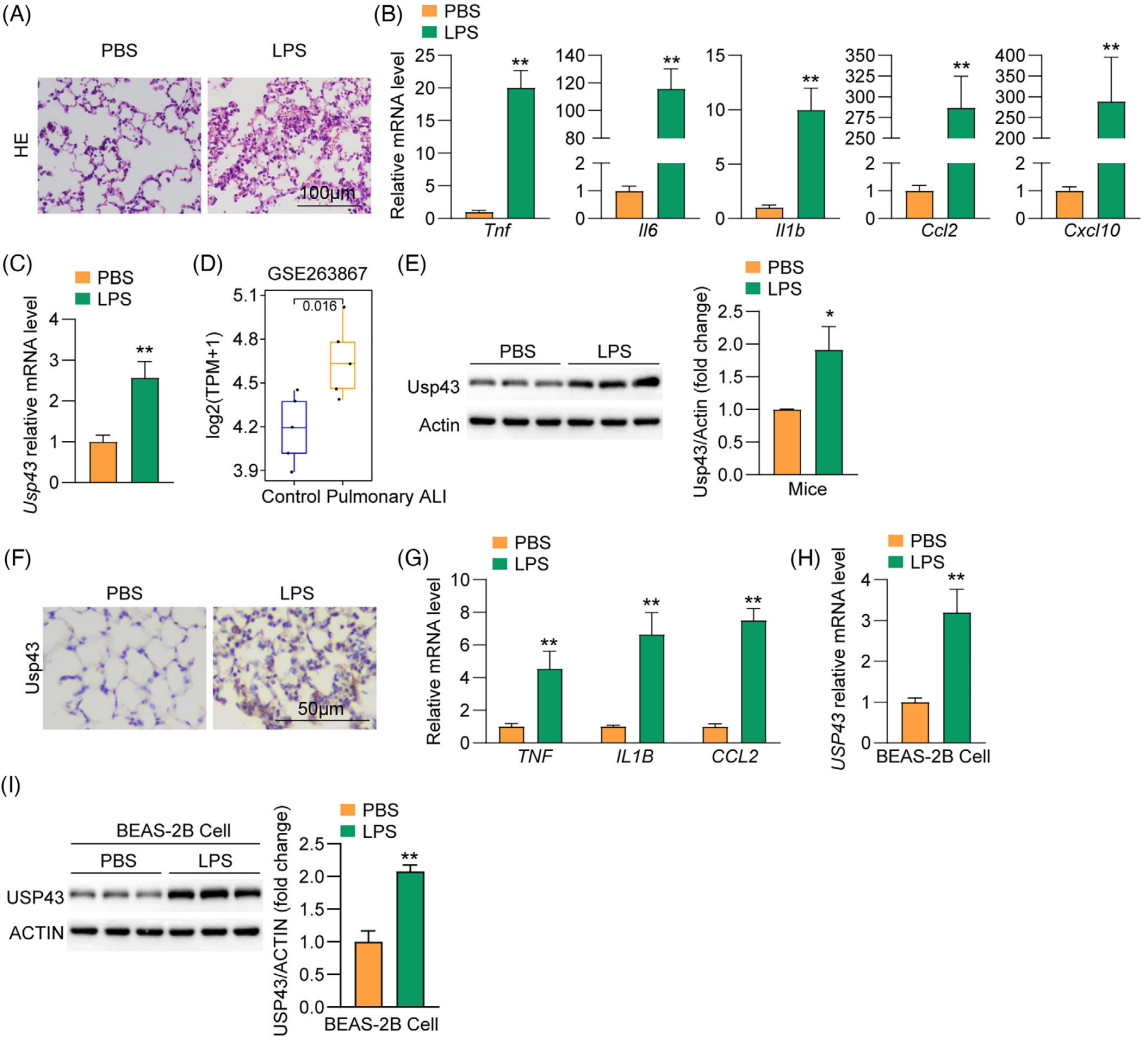
Up‐regulation of ubiquitin‐specific protease 43 (USP43) in lipopolysaccharide (LPS)‐induced ALI. (A) Haematoxylin and eosin (H&E) staining of lung tissues from PBS‐ or LPS‐treated mice (*n* = 5 mice per group). Scale bar, 100 µm. (B) Relative mRNA expression levels of inflammatory cytokines (*Tnf*, *Il6*, *Il1b*, *Ccl2* and *Cxcl10*) in lung tissues of PBS‐ or LPS‐treated mice (*n* = 4 mice per group). (C) Relative mRNA expression of *Usp43* in lung tissues of PBS‐ or LPS‐treated mice (*n* = 4 mice per group). (D) Expression levels of *Usp43* in the pulmonary ALI and control groups from the GSE263867 dataset (GEO Accession viewer, five samples in per group). (E) Western blot analysis (Left) and quantitative result (Right) of Usp43 protein expression in lung tissues of PBS‐ or LPS‐treated mice (*n* = 3 mice per group). (F) Representative image of USP43 immunohistochemical staining of lung tissues from PBS‐ or LPS‐treated mice (*n* = 4 mice per group). Scale bar, 50 µm. (G) Relative mRNA expression levels of inflammatory cytokines (*TNF*, *IL1B*, *CCL2*) in LPS‐treated and control BEAS‐2B cells (*n* = 3 independent biological repetitions). (H) Relative mRNA expression of *USP43* in LPS‐treated and control BEAS‐2B cells (*n* = 3 independent biological repetitions). (I) Western blot analysis (Left) and quantitative result (Right) of USP43 protein expression in LPS‐treated and control BEAS‐2B cells (*n* = 3 independent biological repetitions). For B, C, E, G–I, a Student's *t*‐test was used for statistical analysis. *p < 0.05, **p <0.01 versus PBS group.

To initially investigate the expression of USP43 in ALI, the C57BL/6J mice were stimulated with LPS to establish an ALI model. H&E staining showed characteristic pathological features, including alveolar wall thickening, inflammatory cell infiltration and haemorrhage (Figure 1A), confirming successful LPS‐induced lung injury. Consistent with the ALI phenotype, LPS treatment markedly increased mRNA levels of pro‐inflammatory cytokines (*Tnf*, *Il6*, *Il1b*, *Ccl2* and *Cxcl10*) in lung tissues (Figure 1B). Importantly, *Usp43* mRNA expression was significantly up‐regulated in ALI mice lung tissues compared to controls (Figure 1C). Subsequently, we analysed the data from the Control group and the pulmonary ALI group (intratracheally injected) in the GSE263867 dataset. It has been reported in the relevant literature of this dataset that principal component analysis shows that there is a significant clustering in the Dim1 dimension between the Control group and the pulmonary ALI group. The GO and KEGG analyses revealed that the inflammatory‐related pathways were significantly activated in the pulmonary ALI group.^21^ Consistent with our result, the expression of *Usp43* was up‐regulated in the pulmonary ALI group of GSE263867 dataset (Figure 1D). Subsequently, we examined the protein expression of Usp43 and found that the expression level of Usp43 in the lung tissues of the ALI group of mice was significantly higher than that of the control group (Figure 1E). Moreover, immunohistochemical staining also revealed that the positive signal of Usp43 was enhanced in the lung tissues of mice in the LPS administered group (Figure 1F). Furthermore, we used LPS to treat epithelial cells in vitro. LPS treatment elevated inflammatory cytokine expression (*TNF*, *IL1B*, *CCL2*; Figure 1G). USP43 was up‐regulated both in mRNA level (Figure 1H) and in protein level (Figure 1I). These consistent findings across murine and cellular ALI models suggest that USP43 is up‐regulated during the ALI process, and it may play a potential role in the pathogenesis of ALI.

### 3.2 USP43 deficiency aggravates ALI in mice

**FIGURE 2:**
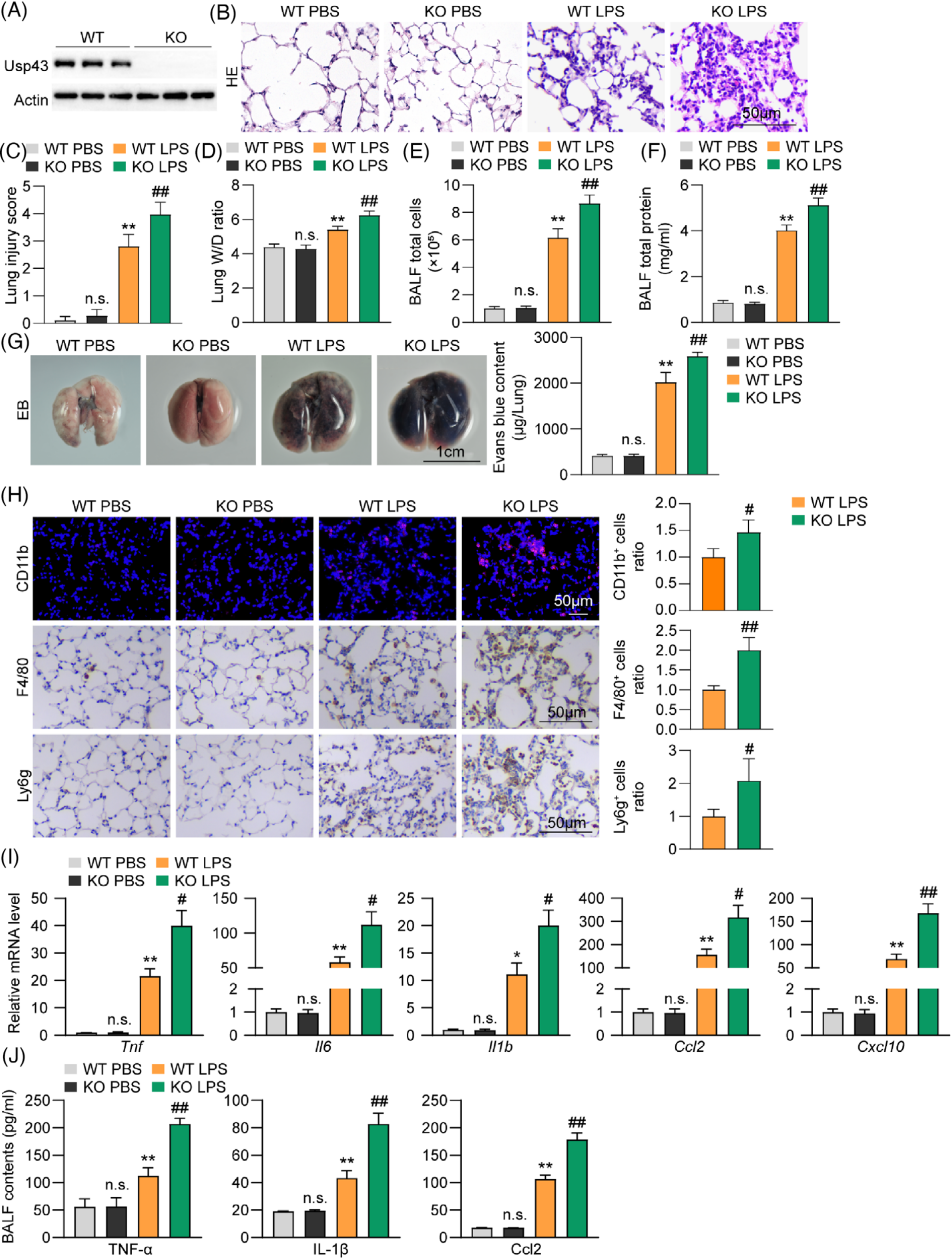
Ubiquitin‐specific protease 43 (USP43) knockout aggravates lipopolysaccharide (LPS)‐induced ALI in mice. (A) Western blot validation of Usp43 knockout efficiency in *Usp43*‐KO mice (*n* = 3 mice per group). (B) Representative haematoxylin and eosin (H&E)‐stained lung tissue images from mice in different groups (*n* = 6 mice per group). Scale bar, 50 µm. (C) Lung injury scores in the indicated groups (*n* = 6 mice per group). (D) Lung wet/dry weight ratios in the indicated groups (*n* = 8 mice per group). (E) The total cell content in the bronchoalveolar lavage fluid (BALF) of each group of mice (*n* = 8 mice per group). (F) The detection results of total protein concentration in the BALF of each group of mice (*n* = 8 mice per group). (G) Representative images of Evans Blue staining (Left) and detection analysis of Evans Blue extravasation (Right) in lung tissues (*n* = 6 mice per group). Scale bar, 1 cm. (H) Representative images of immune cells immunohistochemical and immunofluorescence staining (Left) in lung tissues and quantitative analysis (Right) results (*n* = 4 mice per group). Scale bar, 50 µm. (I) Relative mRNA expression levels of inflammatory cytokines (*Tnf*, *Il6*, *Il1b*, *Ccl2* and *Cxcl10*) in lung tissues of mice from the indicated groups (*n* = 4 mice per group). (J) The enzyme‐linked immunosorbent assay (ELISA) test results for the content of inflammatory cytokines (TNF‐α, IL‐1β and Ccl2) in BALF (*n* = 6 mice per group). For C–J, the one‐way ANOVA analysis followed by Bonferroni's post‐hoc test or Tamhane's T2 (M) post‐hoc test was used for statistical analysis. *p < 0.05, **p < 0.01 versus wild‐type (WT) PBS group. #*p* < 0.05, ##*p* < 0.01 versus WT LPS group. n.s., no significance versus WT PBS group.

To investigate the role of USP43 in ALI pathogenesis in vivo, LPS was administered via intratracheal instillation to *Usp43* KO mice and WT littermate controls (Figure 2A). No phenotypic differences were observed between genotypes under basal conditions. Histopathological analysis of H&E‐stained lung sections revealed exacerbated pulmonary damage in *Usp43*‐KO mice post‐LPS challenge compared to WT controls, characterised by prominent inflammatory infiltration and marked alveolar wall thickening (Figure 2B). Quantitative lung injury scores were significantly elevated in KO LPS mice versus WT LPS counterparts (Figure 2C). Usp43 deficiency intensified LPS‐induced pulmonary oedema, as indicated by increased lung W/D ratios (Figure 2D). BALF analysis demonstrated elevated total cell counts (Figure 2E) and protein content (Figure 2F) in KO LPS mice, indicating USP43 loss potentiates LPS‐induced alveolar–capillary barrier disruption. Evans Blue extravasation assays confirmed enhanced vascular permeability in KO mice (Figure 2G). Immunofluorescence staining revealed augmented CD11b^+^ myeloid cell infiltration in KO LPS lungs (Figure 2H). Immunohistochemical staining showed that the absence of Usp43 significantly promoted the infiltration of Ly6G‐positive neutrophils and F4/80‐positive macrophages stimulated by LPS (Figure 2H). Reverse transcription‐polymerase chain reaction (RT‐PCR) analysis showed Usp43 ablation amplified LPS‐triggered up‐regulation of pro‐inflammatory genes (*Tnf*, *Il6*, *Il1b*, *Ccl2*, *Cxcl10*; Figure 2I). Consistently, BALF cytokine (TNF‐α, IL‐1β, Ccl2) concentrations were highest in KO LPS group (Figure 2J). Collectively, these result indicated USP43 deficiency exacerbates the progression of ALI.

### 3.3 USP43 negatively regulates LPS‐induced epithelial cell injury and inflammatory responses in vitro

**FIGURE 3:**
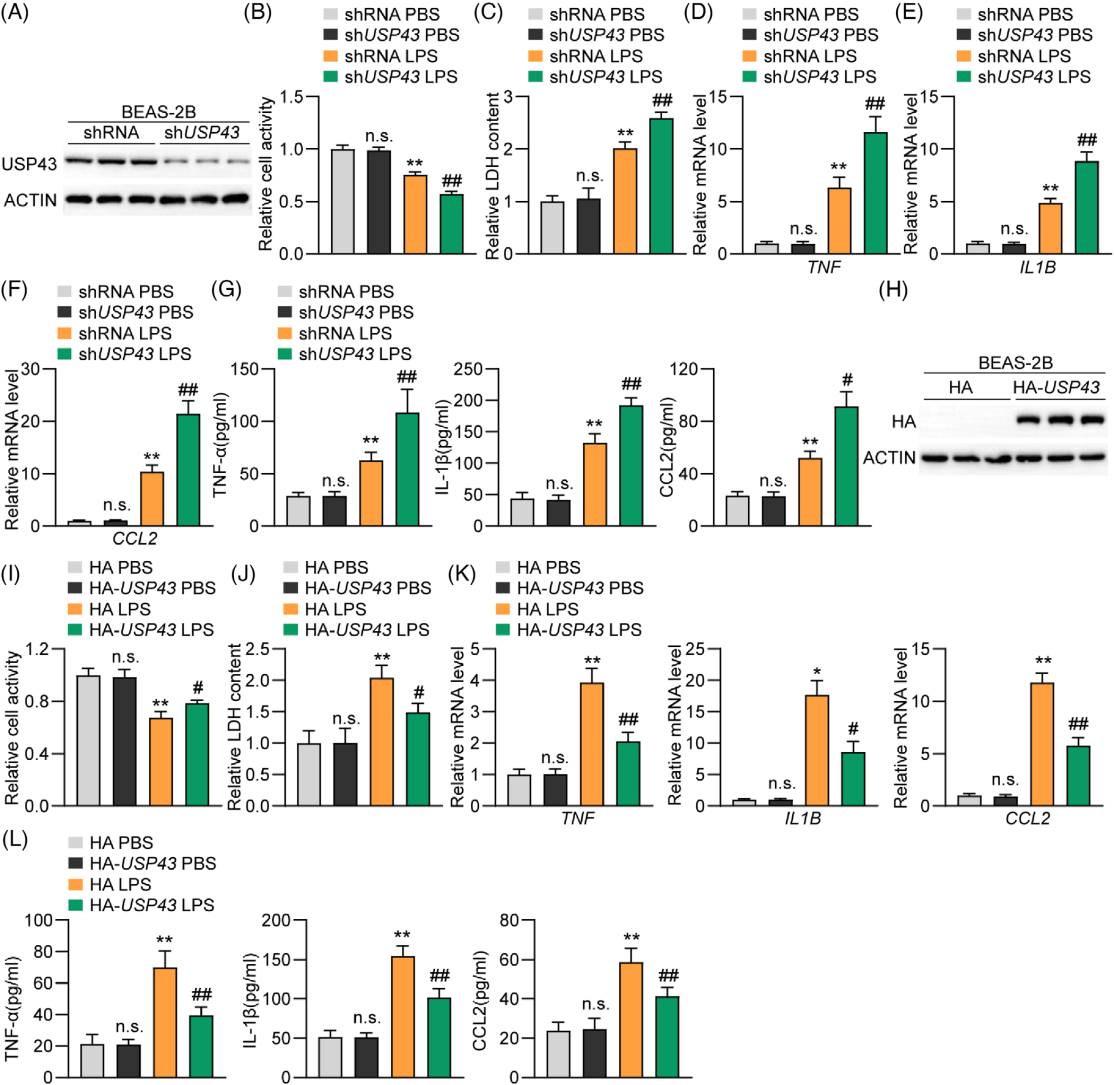
Ubiquitin‐specific protease 43 (USP43) modulates lipopolysaccharide (LPS)‐induced cell injury and inflammatory responses in BEAS‐2B cells. (A) Western blot detection results of USP43 in USP43‐knockdown (sh*USP43*) and control (shRNA) BEAS‐2B cells. (B) The CCK8 assay results for cell viability of sh*USP43* and shRNA cells treated with PBS or LPS. (C) Relative LDH content detection results of sh*USP43* and shRNA cells treated with PBS or LPS. (D–F) Relative mRNA expression of inflammatory cytokines (*TNF* (D), *IL1B* (E) and *CCL2* (F)) in sh*USP43* and shRNA cells after PBS or LPS treatment. (G) Enzyme‐linked immunosorbent assay (ELISA) quantification of secreted inflammatory cytokines (TNF‐α, IL‐1β, CCL2) in the medium of sh*USP43* and shRNA cells after PBS or LPS treatment. (H) Western blot detection results of USP43 in USP43‐overexpression (HA‐*USP43*) and control (HA) BEAS‐2B cells. (I) The CCK8 assay results for cell viability of HA‐*USP43* and control cells treated with PBS or LPS. (J) Relative LDH content detection results of HA‐*USP43* and control cells treated with PBS or LPS. (K) Relative mRNA expression of inflammatory cytokines (*TNF*, *IL1B* and *CCL2*) in HA‐USP43 and control cells after PBS or LPS treatment. (L) ELISA quantification of secreted inflammatory cytokines (TNF‐α, IL‐1β, CCL2) in the medium of HA‐*USP43* and control cells after PBS or LPS treatment. *n* = 3–4 independent biological repetitions. The one‐way ANOVA test followed by Bonferroni's post‐hoc test or Tamhane's T2 (M) post‐hoc test was used for statistical analysis. n.s., no significance versus shRNA PBS group or HA PBS group. *p < 0.05, **p < 0.01 versus shRNA PBS group or HA PBS group. #*p* < 0.05, ##*p* < 0.01 versus shRNA LPS group or HA LPS group.

Epithelial cells exert vital role in maintaining lung homeostasis and are closely implicated in the pathophysiological process of ALI.^22^, ^23^ To further confirm the regulatory role of USP43 in epithelial cells, we constructed a USP43 knockdown BEAS‐2B cells line (sh*USP43*; Figure 3A). In the PBS treatment group, the knockdown of USP43 had no significant effect on cell activity and LDH production. However, following LPS stimulation, the knockdown of USP43 significantly promoted the decrease in cell activity and the release of LDH (Figure 3B,C). In addition, USP43 knockdown promoted LPS‐induced inflammatory response, as demonstrated by elevated mRNA expression of pro‐inflammatory cytokines (*TNF*, *IL1B* and *CCL2*; Figure 3D–F), and enhanced cytokine secretion (TNF‐α, IL‐1β and CCL2; Figure 3G). Furthermore, the USP43 overexpressed BEAS‐2B cell line was successfully constructed (Figure 3H). After LPS stimulation, USP43 overexpression exhibited protective effects, partially reversing the LPS‐induced cell injury (Figure 3I,J) and attenuating inflammatory responses (Figure 3K,L).

To further verify the function of USP43 in alveolar epithelial cells, we constructed a Usp43 knockdown MLE12 cell line and subjected it to LPS stimulation (Figure S1A). The results consistently demonstrated that knockdown of Usp43 could promote LPS‐induced cell damage and inflammatory activation in MLE12 cells (Figure S1B–F). Subsequently, it was also observed that USP43 exhibited the same protective effect in the USP43‐overexpressing MLE12 cell line as it did in the BEAS‐2B cells (Figure S1G–L).

These findings are consistent with the results in vivo, which collectively demonstrate that USP43 serves as a negative regulator of LPS‐induced cell injury and inflammatory responses during ALI.

### 3.4 USP43 regulates LPS‐induced ferroptosis in vivo and in vitro

**FIGURE 4:**
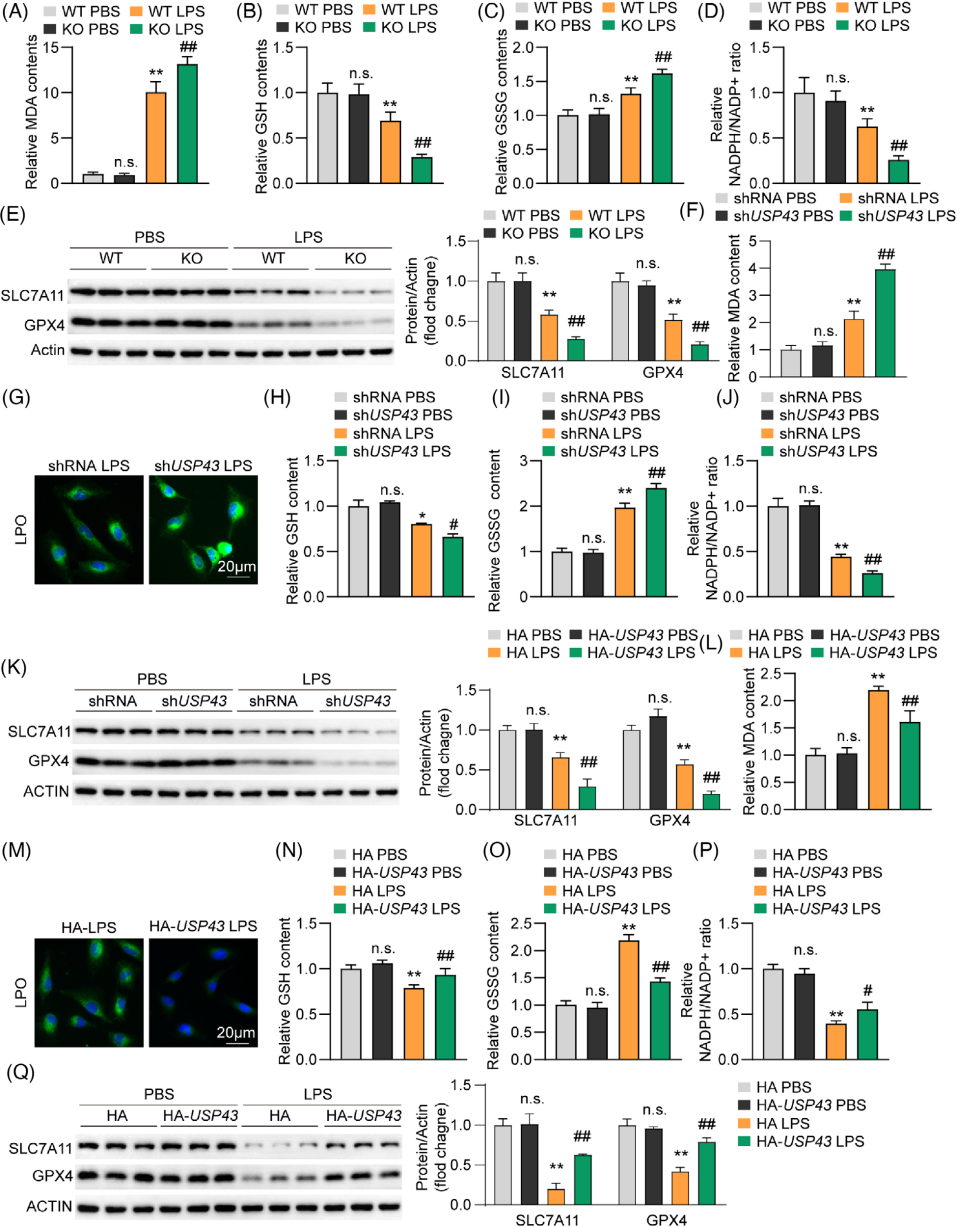
Ubiquitin‐specific protease 43 (USP43) knockdown exacerbates while its overexpression alleviates lipopolysaccharide (LPS)‐induced ferroptosis. (A–D) Relative malondialdehyde (MDA) contents (A), glutathione (GSH) contents (B), oxidised glutathione (GSSG) contents (C) and NADPH/NADP^+^ ratio (D) in lung tissues of wild type (WT) and *Usp43*‐KO mice after PBS or LPS treatment (*n* = 6 mice per group). (E) Western blot analysis (Left) and quantitative result (Right) of solute carrier family 7 member 11 (SLC7A11) and GPX4 protein expression in mouse lung tissues of the indicated group (*n* = 3 mice per group). (F) Relative MDA contents in USP43‐knockdown and control BEAS‐2B cells after PBS or LPS treatment (*n* = 4 independent biological repetitions). (G) Representative immunofluorescence image of lipid peroxidation staining using the BODIPY 581/591 C11 kit (*n* = 3 independent biological repetitions). (H‐J) Relative GSH contents (H), GSSG contents (I) and NADPH/NADP^+^ ratio (J) in USP43‐knockdown and control BEAS‐2B cells after PBS or LPS treatment (*n* = 4 independent biological repetitions). (K) Western blot analysis (Left) and quantitative result (Right) of SLC7A11 and GPX4 protein levels in cells with the indicated treatment (*n* = 3 independent biological repetitions). (L) Relative MDA contents in USP43‐overexpression and control cells after PBS or LPS treatment (*n* = 4 independent biological repetitions). (M) Representative immunofluorescence image of lipid peroxidation staining using the BODIPY 581/591 C11 kit (*n* = 3 independent biological repetitions). (N–P) Relative GSH contents (N), GSSG contents (O) and NADPH/NADP^+^ ratio (P) in USP43‐ overexpression and control cells after PBS or LPS treatment (*n* = 4 independent biological repetitions). (Q) Western blot analysis (Left) and quantitative result (Right) of SLC7A11 and GPX4 protein levels in cells with the indicated treatment (*n* = 3 independent biological repetitions). The one‐way ANOVA test followed by Bonferroni's post‐hoc test or Tamhane's T2 (M) post‐hoc test was used for statistical analysis. n.s., no significance versus WT PBS group or shRNA PBS group or HA PBS group. *p < 0.05, **p < 0.01 versus WT PBS group or shRNA PBS group or HA PBS group. #*p* < 0.05, ##*p* < 0.01 versus WT LPS group or shRNA LPS group or HA LPS group.

Ferroptosis has been proven to be an important mechanism for the development of ALI.^7^, ^24^ In addition, previous studies have confirmed that USP43 is involved in the regulation of ferroptosis.^15^ Based on these backgrounds, we reasonably explored the regulatory effect of USP43 on ferroptosis during LPS‐induced ALI. Based on the significant role of labile iron pools in ferroptosis, we first investigated the effect of changes in USP43 expression on the intracellular Fe^2+^ content in cells after LPS treatment. The results indicated that knockdown or overexpression of USP43 did not affect the increase of Fe^2+^ content in cells stimulated by LPS (the result was not shown). Then we further focused our research on the lipid peroxidation process rather than iron metabolism. We examined oxidative stress markers in PBS‐ or LPS‐treated mice and cells. The absence of Usp43 significantly exacerbated the production of lipid peroxides caused by LPS stimulation (Figure 4A). Furthermore, Usp43 KO mice showed decreased GSH content (Figure 4B), elevated GSSG content (Figure 4C), and reduced NADPH/NADP^+^ ratio (Figure 4D) following LPS treatment. Regarding the SLC7A11‐GPX4 axis serves as the key antioxidant pathway to inhibit lipid peroxide accumulation. A large number of published studies have routinely selected these two representative markers to evaluate ferroptosis activation.^25^, ^26^ Therefore, we primarily detected these two pivotal proteins to reflect the lipid peroxidation status in this study. Western blot analysis revealed that Usp43 deficiency markedly promoted the down‐regulation of the ferroptosis‐related molecules SLC7A11 and GPX4 in lung tissue in response to LPS stimulation (Figure 4E).

Consistently, in LPS‐stimulated BEAS‐2B cells, USP43 knockdown also increased the content of lipid peroxidation, reduced the antioxidant capacity of the cells (Figure 4F–J). After LPS stimulation, the protein contents of GPX4 and SLC7A11 in the USP43 knockdown group were also significantly lower than those in the control group (Figure 4K). Conversely, USP43 overexpression had the opposite effect, as demonstrated by reduced lipid peroxidation, enhanced antioxidant activity, and restored SLC7A11 and GPX4 levels (Figure 4L–Q). In addition, the same results were also observed in MLE12 cells (Figure S2). Overall, these consistent results both in vivo and in vitro demonstrated that USP43 inhibits ferroptosis during ALI.

### 3.5 USP43 regulates ALI progression major through influencing ferroptosis

**FIGURE 5:**
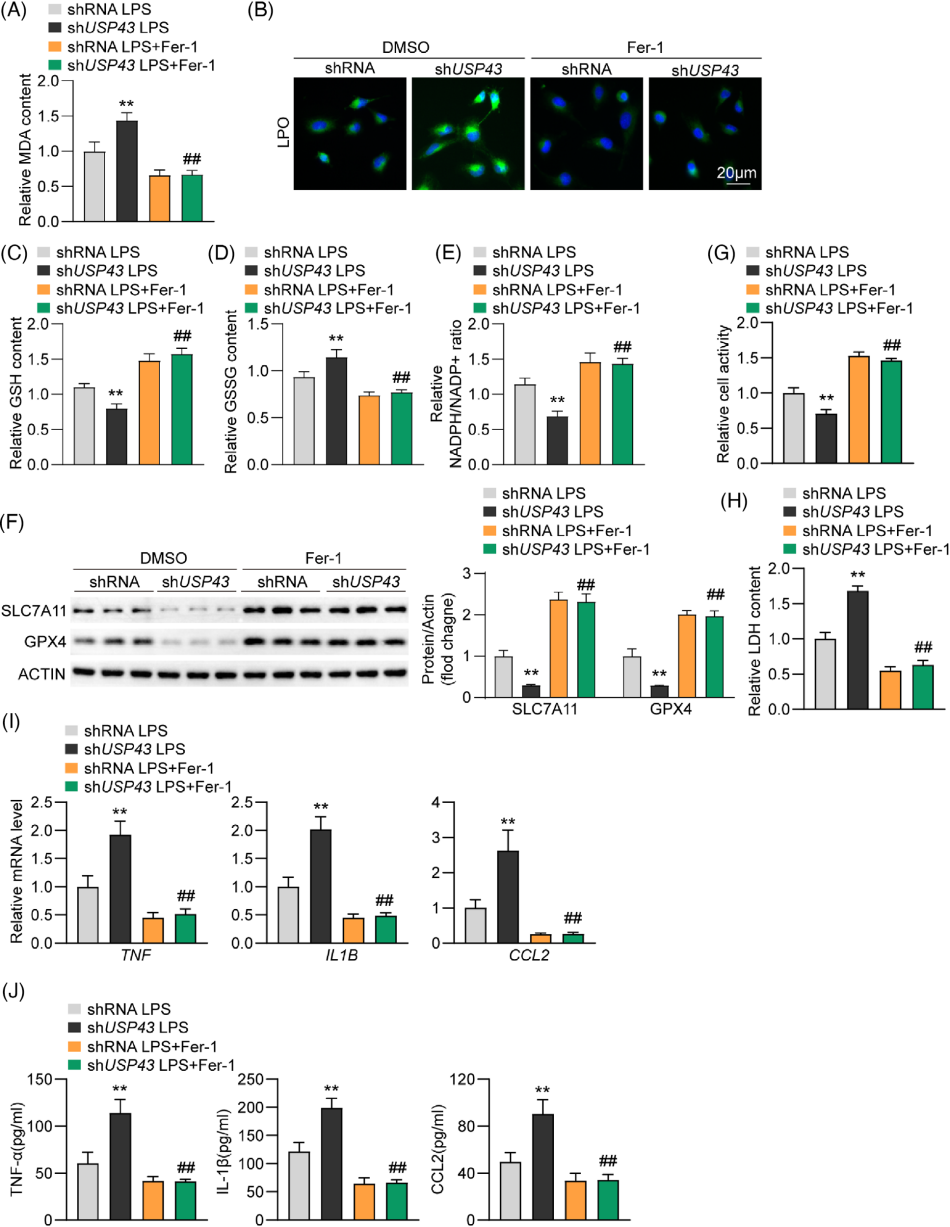
Fer‐1 treatment reversed the promoting effects of ubiquitin‐specific protease 43 (USP43) knockdown on lipopolysaccharide (LPS)‐induced ferroptosis, cell damage and inflammation. (A) Relative malondialdehyde (MDA) contents detection result in USP43 knockdown and control cells after Fer‐1 pretreatment and stimulated by LPS (*n* = 4 independent biological repetitions). (B) Representative immunofluorescence image of lipid peroxidation staining using the BODIPY 581/591 C11 kit in the indicated groups (*n* = 3 independent biological repetitions). (C–E) Relative glutathione (GSH) contents (C), oxidised glutathione (GSSG) contents (D) and NADPH/NADP^+^ ratio (E) in USP43 knockdown and control cells after Fer‐1 pretreatment and stimulated by LPS (*n* = 4 independent biological repetitions). (F) Western blot analysis (Left) and quantitative result (Right) of solute carrier family 7 member 11 (SLC7A11) and GPX4 protein expression in the indicated cells (*n* = 3 independent biological repetitions). (G) Relative cell activity assessed by CCK‐8 assay in the indicated groups (*n* = 3 independent biological repetitions). (H) Relative content of LDH in the medium of the indicated groups (*n* = 3 independent biological repetitions). (I) Relative mRNA expression of inflammatory cytokines (*TNF*, *IL1B* and *CCL2*) in cells from the indicated group (*n* = 4 independent biological repetitions). (J) Enzyme‐linked immunosorbent assay (ELISA) quantification of secreted inflammatory cytokines (TNF‐α, IL‐1β, CCL2) in cell culture supernatants of the indicated group (*n* = 4 independent biological repetitions). The one‐way ANOVA test followed by Bonferroni's post‐hoc test or Tamhane's T2 (M) post‐hoc test was used for statistical analysis. *p < 0.05, **p < 0.01 versus shRNA LPS group. #*p* < 0.05, ##*p* < 0.01 versus sh*USP43* LPS group.

To determine whether the regulation of ALI by USP43 associates with its role in ferroptosis, we treated USP43 knockdown cells and their control cells with the ferroptosis inhibitor Fer‐1 and then stimulated them with LPS. Fer‐1 treatment significantly abolished the increase of lipid peroxides and the reduction of antioxidant capacity caused by USP43 knockdown in response to LPS stimulation (Figure 5A–E). Fer‐1 also reversed the down‐regulation of SLC7A11 and GPX4 in USP43 knockdown group (Figure 5F). This indicated that the Fer‐1 treatment eliminated the promoting effect of USP43 knockdown on ferroptosis. Furthermore, as shown in the results of CCK8 and LDH assays, Fer‐1 treatment abolished the reduction in cell viability and the increase in cell damage caused by USP43 knockdown in response to LPS stimulation (Figure 5G,H). In terms of the inflammatory response, Fer‐1 also reversed the increase in the expression and secretion of inflammatory‐associated factors TNF‐α, IL‐1β and CCL2 caused by the knockdown of USP43 (Figure 5I,J).

Subsequently, Erastin, a ferroptosis inducer, was used to treat BEAS‐2B cells. The results showed that the generation of lipid peroxides and the decline in antioxidant capacity induced by Erastin were both alleviated by the overexpression of USP43 (Figure S3A–E). Additionally, USP43 overexpression also significantly relieved the cell damage and inflammation activation caused by Erastin treatment (Figure S3F–H). Together, our results demonstrated that the regulatory effect of USP43 on ALI major depends on ferroptosis.

### 3.6 USP43 stabilises SLC7A11 by removing K48‐linked ubiquitination

**FIGURE 6:**
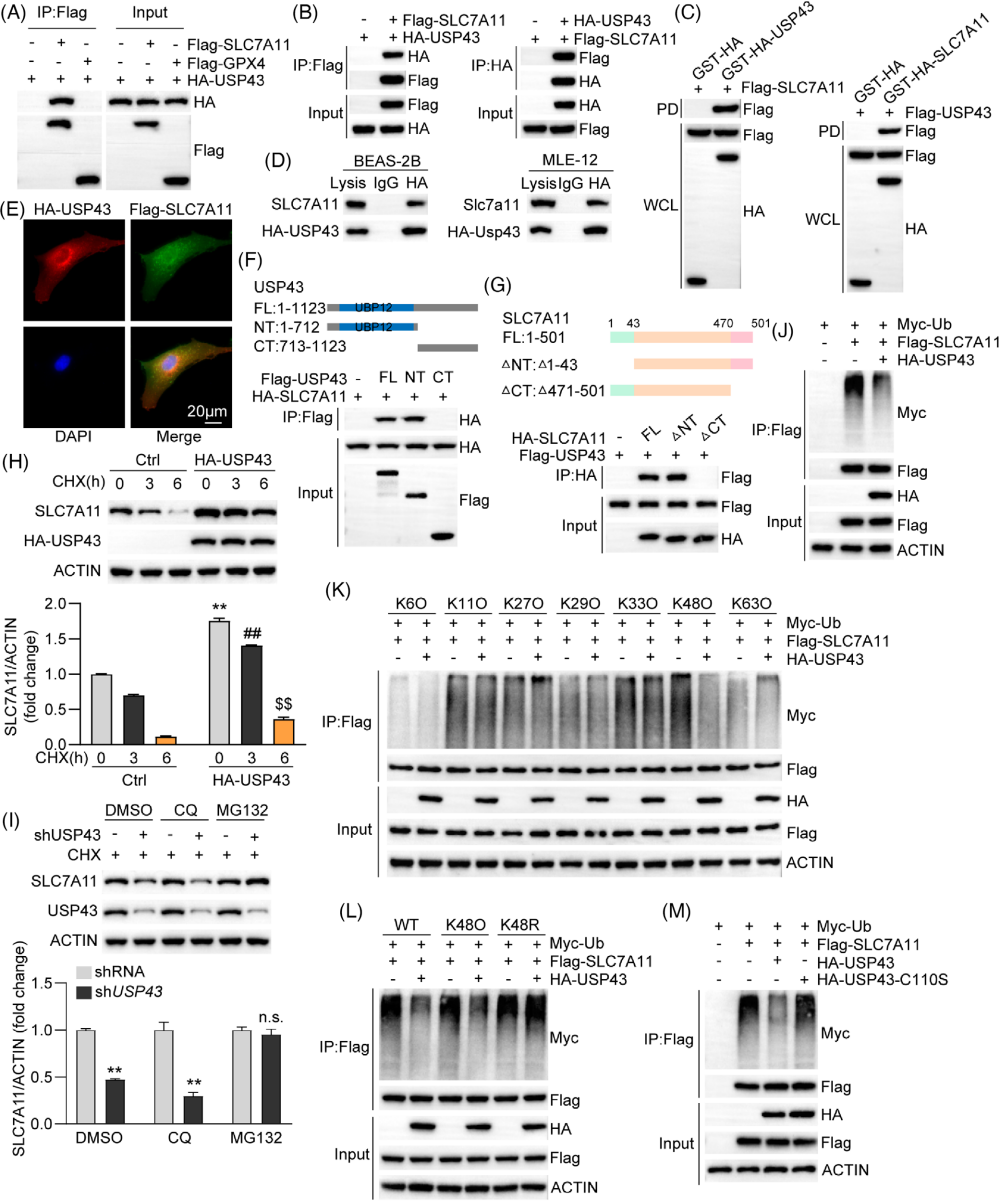
Ubiquitin‐specific protease 43 (USP43) directly interacts with and stabilises solute carrier family 7 member 11 (SLC7A11) through removing K48‐linked ubiquitination. (A) The results of Western blot analysis of the proteins obtained after co‐immunoprecipitation (Co‐IP) of the cell lysates of HEK293T cells that were overexpressed with USP43, SLC7A11 and GPX4 respectively or simultaneously. (B) The results of Western blot analysis of the proteins obtained after Co‐IP of the cell lysates of HEK293T cells that were overexpressed with USP43 and SLC7A11 respectively or simultaneously. (C) GST pull‐down assay results for direct binding between USP43 and SLC7A11 in HEK293T cells. (D) Hemi‐endogenous Co‐IP analysis results of USP43 and SLC7A11 in BEAS‐2B and MLE12 cells. (E) Representative images of immunofluorescence analysis results of USP43 and SLC7A11 co‐localisation in BEAS‐2B cells. (F) Co‐IP analysis results of the interaction domain between HA‐SLC7A11 and Flag‐USP43 truncated mutants. (G) Co‐IP analysis results of the interaction domain between Flag‐USP43 and HA‐SLC7A11 truncated mutants. (H) The Western blot detection and quantitative results of SLC7A11 expression level in BEAS‐2B cells that overexpressing HA‐USP43 and treated with CHX for different durations. (I) Western blot analysis and quantitative results of SLC7A11 expression in BEAS‐2B cells that transfected with sh*USP43* and treated with DMSO, MG132 (5 µM), or chloroquine (50 µM) for 6 h. (J) Ubiquitination assay of results SLC7A11 with or without HA‐USP43 overexpression. (K) Ubiquitination analysis of Flag‐SLC7A11 co‐expressed with Myc‐tagged ubiquitin variants (wild type [WT], K6‐, K11‐, K27‐, K29‐, K33‐, K48‐, or K63‐linked) in HEK293T cells, with or without HA‐USP43. (L) Ubiquitination assay of Flag‐SLC7A11 with Myc‐tagged ubiquitin (WT, K48‐, or K48R) in HEK293T cells, with or without HA‐USP43. (M) Ubiquitination analysis of Flag‐SLC7A11 with Myc‐Ub in HEK293T cells co‐transfected with either HA‐USP43 or HA‐USP43‐C110S mutant. *n* = 3 independent biological repetition. For H and I, a Student's *t*‐test was used for statistical analysis. **p < 0.01 versus Ctrl 0 h group (H) or shRNA group (I), ##*p* < 0.01 versus Ctrl 3 h group (H), $$*p* < 0.01 versus Ctrl 6 h group (H).

Given that USP43 is a DUB and it modulates the expression of SLC7A11 and GPX4, we hypothesised that USP43 regulates ferroptosis by stabilising SLC7A11 or GPX4 through its deubiquitination activity. HA‐tagged USP43 and Flag‐tagged SLC7A11 or GPX4 were transfected into HEK293T cells, respectively or simultaneously. After Co‐IP using anti‐Flag antibody, Western blot analysis was performed. The results show that USP43 can interact with SLC7A11 rather than GPX4 (Figure 6A). The subsequent Co‐IP experiments further confirmed the interaction between USP43 and SLC7A11 (Figure 6B). GST‐pulldown analysis confirmed that USP43 can directly bind to SLC7A11 (Figure 6C). Additionally, in BEAS‐2B cells and MLE12 cells that overexpress HA‐tagged USP43, endogenous SLC7A11 was co‐precipitated with USP43 using an anti‐HA antibody, which further confirmed their interaction in epithelial cells (Figure 6D). Immunofluorescence staining demonstrated that USP43 and SLC7A11 have the same cellular localisation in BEAS‐2B cells (Figure 6E). In order to further confirm the interaction domain between USP43 and SLC7A11, we constructed truncated mutant plasmids of USP43 and SLC7A11. Co‐IP assay indicated that the N‐terminal sequences (amino acids [aa] 1–712) of USP43 as well as the C‐terminal sequences of SLC7A11 (aa 471–501) are necessary for the interaction between these two proteins (Figure 6F,G). Furthermore, translational inhibitor CHX was taken to treat BEAS‐2B cells, and the results demonstrated that USP43 overexpression significantly delayed the degradation of SLC7A11 (Figure 6H). We further verified the degradation pathway by treating cells with the proteasome inhibitor MG132 or the lysosome inhibitor CQ. MG132, but not CQ, reversed the reduction in SLC7A11 levels caused by USP43 knockdown, indicating that USP43 increased the protein level of SLC7A11 via inhibiting the proteasomal degradation (Figure 6I).

We then investigated whether USP43 regulates SLC7A11 stability through deubiquitination. After transfecting Flag‐SLC7A11, HA‐USP43 and Myc‐Ub plasmids into HEK293T cells separately or simultaneously, we found that the overexpression of USP43 significantly decreased the ubiquitination level of SLC7A11 (Figure 6J). To determine the specific ubiquitin linkage type, we transfected cells with different ubiquitin mutants and exposed that USP43 selectively decreased K48‐linked ubiquitination of SLC7A11, while other linkage types remained unaffected (Figure 6K,L). In addition, we generated a catalytically inactive USP43 mutant (C110S, cysteine‐serine substitution) and found that the USP43‐C110S mutant failed to reduce the ubiquitination level of SLC7A11, indicating that USP43 stabilises SLC7A11 protein through its deubiquitinating enzyme activity (Figure 6M).

Considering that USP43 can increase the stability of FASN by removing its ubiquitination in ovarian cancer, thereby regulating the transcriptional expression of SLC7A11. In order to verify whether USP43 also regulates the expression of SLC7A11 in ALI in the same way, we knocked down Fasn in the Usp43 overexpressed MLE12 cells and subjected them to LPS stimulation. Subsequently, the mRNA level of *Slc7a11* was detected and the results indicated that knockdown of Fasn has no significant influence on the mRNA level of *Slc7a11* (Figure S4). This result confirmed that FASN does not mediate the regulatory effect of USP43 on SLC7A11 in the context of LPS‐induced ALI. Together, these findings demonstrate that USP43 protects SLC7A11 from proteasomal degradation by reducing K48‐linked ubiquitination of SLC7A11 in ALI.

Finally, the USP43 (C110S) mutant overexpressed BEAS‐2B cell line was successfully constructed and was stimulated with LPS. The results revealed that the protective effect of USP43 overexpression against ferroptosis induced by LPS disappeared due to the mutation of the USP43 deubiquitinating enzyme activity (Figure S5A–E). The up‐regulation of SLC7A11 caused by USP43 overexpression was completely abolished (Figure S5F). Consistently, the protective effect of USP43 overexpression against the cell damage and inflammatory response induced by LPS was also rescued (Figure S5G–I). Totally, these results indicate that USP43 could remove the K48‐linked deubiquitination of SLC7A11 during ALI, and the regulation of ALI by USP43 depends on its ubiquitinase activity.

### 3.7 SLC7A11 critically mediates the regulatory effect of USP43 on ALI

**FIGURE 7:**
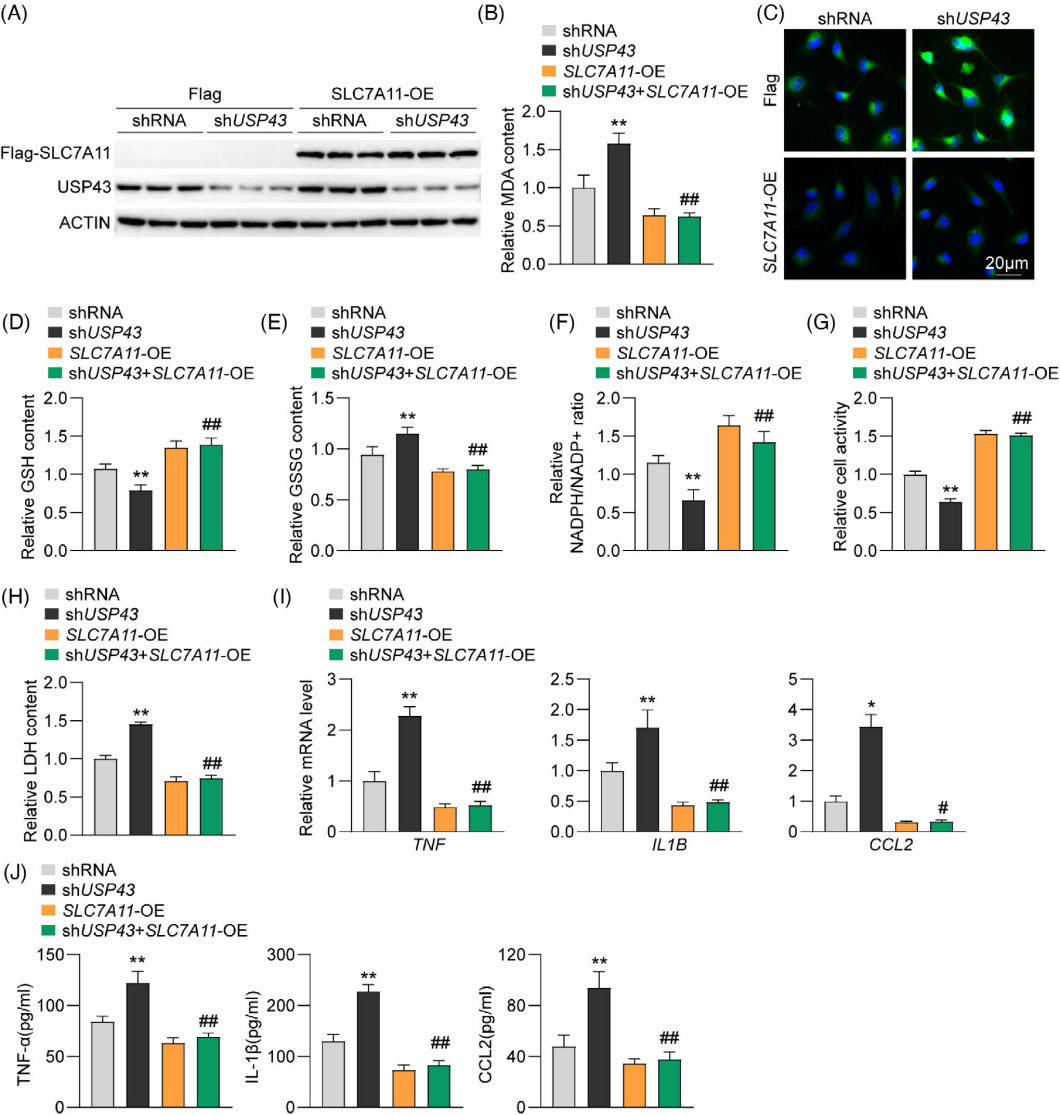
Overexpression of solute carrier family 7 member 11 (SLC7A11) abolished the promoting effect of ubiquitin‐specific protease 43 (USP43) knockdown on lipopolysaccharide (LPS)‐induced epithelial cells. (A) Western blot analysis of Flag‐SLC7A11 and USP43 expression in BEAS‐2B cells with USP43 knockdown and/or SLC7A11 OE (*n* = 3 independent biological repetition). (B) Relative malondialdehyde (MDA) contents in LPS‐treated BEAS‐2B cells with USP43 knockdown and/or SLC7A11 OE (*n* = 4 independent biological repetition). (C) Representative immunofluorescence image of lipid peroxidation staining using the BODIPY 581/591 C11 kit in the indicated groups (*n* = 3 independent biological repetition). (D–F) Relative glutathione (GSH) contents (D), oxidised glutathione (GSSG) contents (E) and NADPH/NADP^+^ ratio (F) in LPS‐treated BEAS‐2B cells with USP43 knockdown and/or SLC7A11 OE (*n* = 4 independent biological repetition). (G) Relative cell activity assessed result by CCK8 assay in the indicated groups (*n* = 4 independent biological repetition). (H) Relative LDH content analysis result in the medium from the indicated groups (*n* = 3 independent biological repetition). (I) Relative mRNA expression of inflammatory cytokines (*TNF*, *IL1B* and *CCL2*) in cells from the indicated groups (*n* = 3 independent biological repetition). (J) Enzyme‐linked immunosorbent assay (ELISA) quantification of secreted inflammatory cytokines (TNF‐α, IL‐1β, CCL2) in cell culture supernatants from the indicated groups (*n* = 4 independent biological repetition). The one‐way ANOVA analysis followed by Bonferroni's post‐hoc test or Tamhane's T2 (M) post‐hoc test was used for statistical analysis. *p < 0.05, **p < 0.01 versus shRNA LPS group. #*p* < 0J.05, ##*p* < 0.01 versus shUSP43 LPS group.

To further confirm whether USP43 exerts its function in regulating the progression of ALI major through SLC7A11, we first overexpressed SLC7A11 in USP43‐knockdown BEAS‐2B cells and then subjected them to LPS stimulation. Western blot assays verified efficient USP43 silencing and successful SLC7A11 overexpression (OE) (Figure 7A). Notably, SLC7A11 overexpression rescued the enhanced ferroptosis induced by USP43 knockdown in response to LPS stimulation (Figure 7B–F). Overexpression of SLC7A11 restored the decreased cell activity and increased LDH release caused by the knockdown of USP43 (Figure 7G,H). Moreover, the supplementation of SLC7A11 significantly eliminated the high inflammatory response caused by USP43 knockdown, which could be demonstrated by the reduction in the production of pro‐inflammatory cytokines (Figure 7I,J). Meanwhile, we knocked down SLC7A11 in the USP43 overexpressing BEAS‐2B cells and then subjected them to LPS stimulation (Figure S6A). The protective effect of USP43 overexpression against ferroptosis, cell damage and inflammatory activation induced by LPS stimulation was abolished when SLC7A11 was knocked down (Figure S6B–I).

**FIGURE 8:**
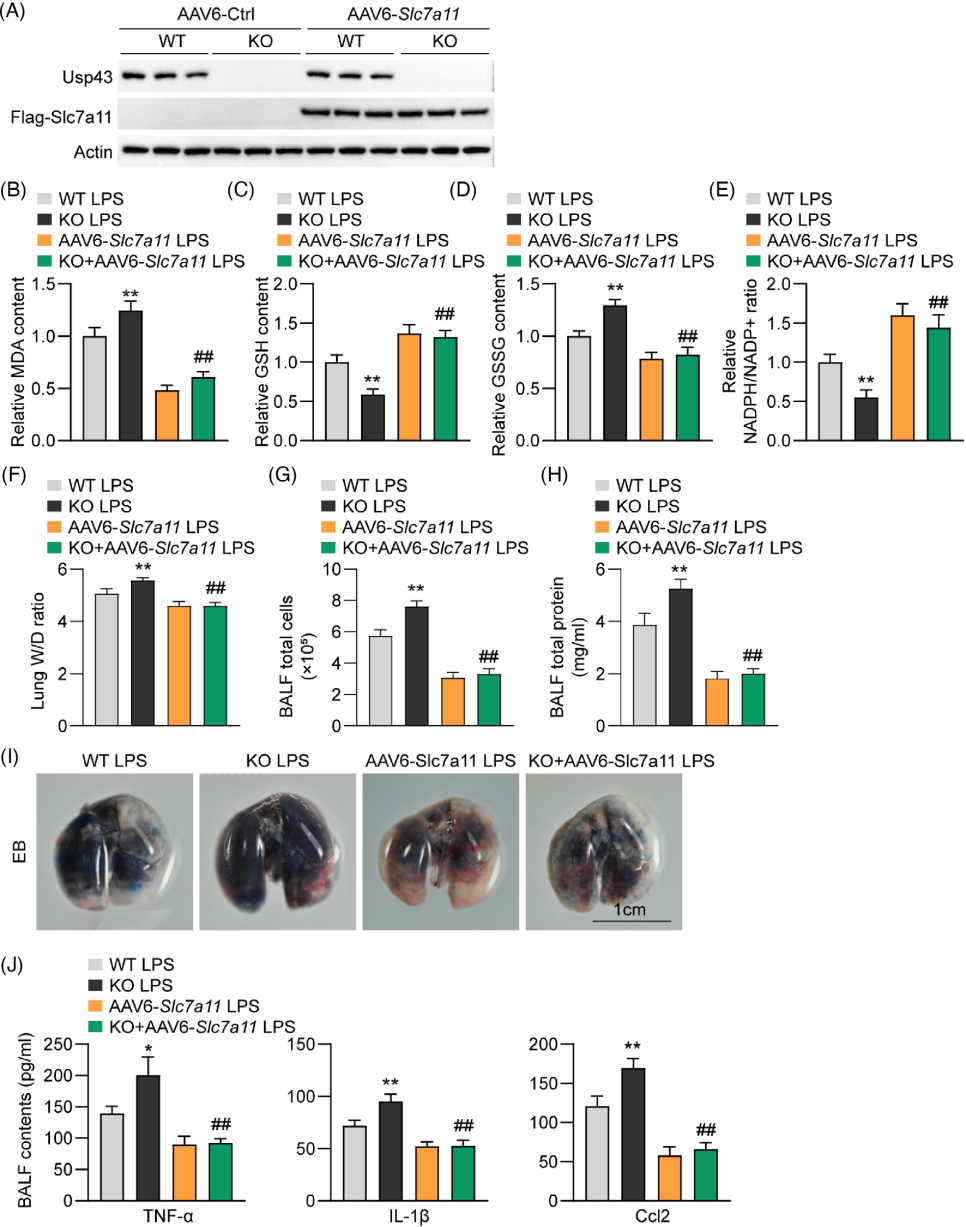
Slc7a11 overexpression reversed the facilitating effect of Usp43 knockout on lipopolysaccharide (LPS)‐induced ALI. (A) Western blot validation of Usp43 knockout and Slc7a11 overexpression efficiency of mice in the indicated group (*n* = 3 mice per group). (B–E) Relative malondialdehyde (MDA) contents (B), glutathione (GSH) contents (C), oxidised glutathione (GSSG) contents (D) and NADPH/NADP^+^ ratio (E) in lung tissues of mice from the indicated group (*n* = 6 mice per group). (F) Lung wet/dry weight ratios in the indicated groups (*n* = 8 mice per group). (G) The total cell content in the bronchoalveolar lavage fluid (BALF) of each group of mice (*n* = 8 mice per group). (H) The detection results of total protein concentration in the BALF of each group of mice (*n* = 8 mice per group). (I) Representative images of Evans Blue staining of lung tissues from the indicated group (*n* = 6 mice per group). Scale bar, 1 cm. (J) The enzyme‐linked immunosorbent assay (ELISA) test results for the content of inflammatory cytokines (TNF‐α, IL‐1β and Ccl2) in BALF of mice from the indicated group (*n* = 6 mice per group). The one‐way ANOVA analysis followed by Bonferroni's post‐hoc test or Tamhane's T2 (M) post‐hoc test was used for statistical analysis. *p < 0.05, **p < 0.01 versus wild‐type (WT) LPS group. #*p* < 0.05, ##*p* < 0.01 versus KO LPS group.

Furthermore, we performed in vivo rescue experiments by intratracheal injection of AAV6‐mediated Slc7a11 overexpression virus into *Usp43*‐KO mice. Western blot results demonstrated the successful overexpression of Slc7a11 in mouse lung tissues (Figure 8A). Phenotypic analysis indicated that Slc7a11 overexpression efficaciously reversed the enhanced ferroptosis induced by LPS in Usp43 KO mice (Figure 8B–E). Importantly, the aggravated lung injury caused by Usp43 deficiency upon LPS challenge was also largely abrogated by Slc7a11 restoration (Figure 8F–J).

Taken together, these data demonstrated that USP43 exerts its protective effects during ALI largely through SLC7A11 as a key downstream mediator both in vitro and in vivo.

## 4 DISCUSSION

ALI/ARDS is a severe clinical condition. It is characterised by an outbreak of acute inflammation, which leads to interstitial oedema of the lung tissue, aggregation of neutrophils and damage to the alveolar epithelium, ultimately resulting in respiratory failure. It is closely related to the high mortality rate in the intensive care field, and there are currently no effective treatment drugs available. The discovery of new potential therapeutic targets for ALI/ARDS is of great significance for the development of drugs for treating ALI/ARDS. In this study, we observed that USP43 expression is up‐regulated during LPS‐induced ALI. However, the biological significance of this up‐regulation remains incompletely understood. One possibility is that USP43 up‐regulation represents a compensatory protective response. The host may attempt to limit excessive ferroptosis and inflammatory injury by enhancing USP43 expression, which in turn stabilises SLC7A11. This interpretation is consistent with our functional data showing that USP43 overexpression alleviates ALI phenotypes. Alternatively, USP43 up‐regulation may simply be an epiphenomenon of cellular stress without a functional adaptive role. Although our gain‐ and loss‐of‐function experiments support the former interpretation, the latter possibility cannot be completely excluded. Future studies examining the temporal dynamics of USP43 expression during the course of ALI may help distinguish between these possibilities.

This study provides evidence for an inhibitory effect of USP43 on ALI based on experiments in bronchial and alveolar epithelial cells and mouse models. Specifically, we found that the absence of USP43 could promote lung injury and inflammatory response caused by LPS stimulation in vivo. In vitro, knockdown of USP43 can also promote LPS‐induced epithelial cell damage and inflammatory activation, while overexpression of USP43 has the opposite effect. Moreover, USP43 exerts its function of inhibiting ALI mainly via suppressing ferroptosis. Mechanistically, USP43 attenuates LPS‐induced ALI mainly by removing K48‐linked polyubiquitination of SLC7A11, thereby stabilising SLC7A11 and suppressing ferroptosis. This study has revealed a new function of USP43 in inhibiting ALI, suggesting that it may represent a potential target for further investigation in ALI/ARDS.

The pathophysiology of ALI involves complex mechanisms, including excessive activation of inflammatory cells, diffuse damage to epithelial cells, pulmonary microvascular endothelial injury and interstitial oedema.^27^ The cytokine storm induces damage to the pulmonary epithelial barrier,^28^ impairing the self‐renewal and differentiation capacity of alveolar type II cells.^29^ In addition, tissue injury disrupts iron homeostasis, leading to excessive intracellular iron accumulation. The labile iron pool catalyses Fenton reactions, generating reactive oxygen species (ROS) that drive uncontrolled lipid peroxidation and ultimately cause ferroptosis.^30^ Targeted regulation of epithelial cell inflammatory and ferroptosis is an important approach for treating ALI.^31^, ^32^, ^33^ In our investigation, we found that USP43 deletion or knockdown can promote the LPS‐induced secretion of inflammatory factors, infiltration of inflammatory cells, production of lipid peroxides, GSSG and NADP^+^, while inhibiting the contents of GSH and NADPH in lung tissues and epithelial cells. However, overexpression of USP43 has the opposite effect. Overall, our results have for the first time indicated that USP43 could regulate the activity and inflammatory response and ferroptosis of bronchial and alveolar epithelial cells during ALI.

Ferroptosis is known to be closely linked to inflammation in various diseases.^34^ Previous studies have shown that intracellular iron overload can activate the NLRP3 inflammasome and increase the level of inflammatory response.^35^, ^36^ Additionally, lipid peroxidation products generated during ferroptosis have been proven to promote the nuclear factor‐κB (NF‐κB) pathway, triggering inflammation in aging kidneys.^37^ The SLC7A11–GSH–GPX4 pathway which plays a critical role in suppressing lipid peroxidation has been found can inhibit NLRP3 inflammasome activation in macrophages.^38^ Moreover, ferroptotic cells also actively release damage‐associated molecular patterns, such as high mobility group box 1 (HMGB1), and then promoting the secretion of inflammatory cytokines.^39^ The ferroptosis inhibitor Fer‐1 has been shown to reduce inflammation in the heart and blood of septic rats,^40^ further supporting that inhibiting ferroptosis alleviates inflammatory responses. Consistent with these observations, our study found that down‐regulation of USP43 led to increased ferroptosis accompanied by higher levels of inflammatory cytokines (TNF‐α, IL‐1β and Ccl2) and more immune cells in murine lungs. In vitro, USP43 attenuated both LPS‐induced ferroptosis and inflammatory responses in epithelial cells. Importantly, when we inhibited ferroptosis with Fer‐1 or overexpressed SLC7A11, the excessive inflammation caused by USP43 deficiency was significantly reduced. These results collectively indicate that USP43 modulates inflammatory outcomes primarily by suppressing ferroptosis.

The SLC7A11–GSH–GPX4 axis is one of the most critical regulatory pathways governing ferroptosis.^41^, ^42^ SLC7A11 is a key component of the cystine/glutamate antiporter system Xc^−^ (composed of SLC7A11 and SLC3A2), which mediates the uptake of extracellular cystine in exchange for intracellular glutamate. The uptaken cystine is rapidly reduced to cysteine and then used for the synthesis of GSH. With the assistance of GSH, GPX4 converts the accumulated lipid hydroperoxides into lipid alcohols, thereby alleviating ferroptosis.^43^, ^44^ In the present study, we experimentally confirmed that USP43 directly interacts with SLC7A11 and inhibits its K48‐linked ubiquitination to maintain its protein stability. Notably, we did not observe a physical interaction between USP43 and GPX4. Although USP43 deficiency also resulted in decreased GPX4 abundance, this alteration may be considered as an indirect secondary change. Existing research has already shown that when the expression of SLC7A11 is down‐regulated or its function is inhibited (such as through Erastin treatment or gene KO), cystine intake decreases, GSH synthesis is insufficient, and this directly leads to a reduction in the protein level of GPX4. Moreover, the insufficient cystine uptake mediated by SLC7A11 can inhibit the transcriptional expression of GPX4 by suppressing the Rag‐mTORC1‐4EBPs signalling pathway.^45^ Therefore, the regulatory effect of USP43 on GPX4 is most likely a passive downstream consequence of SLC7A11 suppression, rather than a direct modulation in this study.

Emerging evidence has established that post‐translational modifications (PTMs) of SLC7A11 serve as sophisticated regulatory mechanisms controlling its protein stability, membrane localisation and transport activity thereby influencing the progression of ferroptosis.^46^, ^47^ These PTMs include but are not limited to ubiquitination,^48^, ^49^ phosphorylation,^50^ palmitoylation,^51^, ^52^ O‐GlcNAcylation,^53^ and SUMOylation,^54^ which collectively form a complex regulatory network. Among these modifications, ubiquitination has emerged as the most prominent and extensively studied regulatory mechanism. E3 ubiquitin–protein ligase TRIM3 has been found to directly interact with SLC7A11 and promotes its K11‐linked ubiquitination and subsequent proteasomal degradation, thereby facilitating ferroptosis in non–small‐cell lung cancer.^55^ The suppressor of cytokine signalling 2 has also been proved to promote K48‐linked polyubiquitin‐mediated degradation of SLC7A11 and ferroptosis in hepatocellular carcinoma.^56^ Conversely, the deubiquitylases such as OTUB1, USP9X and USP14, have been found to contribute to the regulation of ferroptosis by promoting the deubiquitination of SLC7A11.^49^, ^57^, ^58^ All of these indicate that SLC7A11 is an effective and important target for regulating ferroptosis. This study found that USP43 can interact with SLC7A11 and remove the K48‐linked ubiquitination and degradation of SLC7A11, thereby inhibiting ferroptosis during the ALI process. This is consistent with the function of USP43 as a deubiquitinating enzyme. Additionally, studies have shown that USP43 does not interact with SLC7A11 in ovarian cancer. Its regulation on SLC7A11 is achieved indirectly by stabilising the expression of FASN, which is different from our research results.^15^ However, we found that USP43 does not regulate SLC7A11 in a manner dependent on FASN in ALI. The reason for this difference might be attributed to the different types of diseases being studied and the different cell types involved. Ovarian cancer cells exhibit unique lipid metabolic features, with high expression of FASN, a key enzyme in fatty acid synthesis.^59^ Thus, USP43 may preferentially regulate SLC7A11 through FASN in this context. In contrast, in our LPS‐induced ALI model, the metabolic environment differs substantially from that of tumour cells, and USP43 directly target SLC7A11. Second, variations in protein expression levels and interaction networks may lead to different regulatory patterns. The binding affinity of USP43 for SLC7A11 or FASN may differ across cell types, or tissue‐specific competing interactors may exist. Moreover, distinct stress conditions may influence USP43 substrate selectivity. Future studies across different cell types and disease models are needed to elucidate the determinants of USP43 substrate selectivity.

Our research still has some limitations. Firstly, although we have identified an up‐regulation of USP43 expression in ALI, the specific upstream regulatory mechanism remains unclear. Previous studies have demonstrated that YY1 promotes USP43 transcription in ovarian cancer,^15^ and that ETS1 participates in its transcriptional regulation in hepatocellular carcinoma.^60^ Nevertheless, whether these transcription factors directly regulate USP43 transcription during ALI remains an open question that warrants further investigation. Secondly, our investigation has demonstrated that USP43 can inhibit the LPS‐induced inflammatory response in lung tissues and epithelial cells. Furthermore, we also found that regulating ferroptosis can rescue the effect of USP43 on the LPS‐stimulated inflammatory response. This indicates that the regulation of inflammation by USP43 is mainly achieved through its effect on ferroptosis. However, the current data cannot distinguish whether USP43 has a direct regulatory effect on the activation of inflammatory signalling pathway or the infiltration of immune cells. Thirdly, we did not focus on whether USP43 has effects on apoptosis, necroptosis or pyroptosis in the overall study, which makes it difficult to completely exclude the potential contributions of other cell death pathways to the USP43‐mediated phenotypic changes. Fourthly, there are many other types of cells in the lung tissue, including macrophages and endothelial cells. Our research merely demonstrated the regulation of USP43 on bronchial epithelial cells and alveolar epithelial cells during the process of ALI. Further investigation is needed to determine whether USP43 exerts similar functions in endothelial cells or other lung cell types during ALI. Additionally, further validation in clinical samples would strengthen the translational implications in future studies. Finally, the correlation between USP43 and SLC7A11 expression and clinical outcomes in ALI patients requires further exploration.

## 5 CONCLUSION

This study is the first time to demonstrate that USP43 inhibits ferroptosis and inflammatory responses in bronchial and alveolar epithelial cells by mediating K48‐linked deubiquitination of SLC7A11, suggesting a protective role in ALI. These findings identify the USP43‐SLC7A11‐ferroptosis axis as a potential mechanistic node for further investigation in sepsis‐associated ALI.

## AUTHOR CONTRIBUTIONS

Li Zhang and Jutong He designed the study, conducted animal and molecular biology experiments, wrote and revised the manuscript. Ming Xu provided overall guidance and funding support. Bamawa Mwengendi Joël and Guiomar Correia conducted cell experiment, and analysed experimental data. Xinyi Li carried out project management and provided experimental materials. Xuefeng Zhou and Hexiao Tang conceived the study and provided funding support.

## CONFLICT OF INTEREST STATEMENT

The authors declare no conflicts of interest.

## ETHICS STATEMENT

The animal experiment was approved by the Animal Ethics Committee of Zhongnan Hospital of Wuhan University (approval number ZN2025174). We confirm that all experiments were performed in accordance with relevant named guidelines and regulations.

## CONSENT

The authors have nothing to report.

## Supporting information



Supporting Information

Supporting Information

## Data Availability

The data can be obtained from the corresponding author upon request.
