# Supplementary material for: USP43‐mediated deubiquitination of SLC7A11 protects against LPS‐induced acute lung injury by inhibiting ferroptosis
Source: Clin Transl Med. 2026 Jun 30;16(7):e70718. doi: 10.1002/ctm2.70718 (PMC13319409; doi:10.1002/ctm2.70718)
Supplement: Supplementary file 1 — Supporting Information [file CTM2-16-e70718-s002.docx]

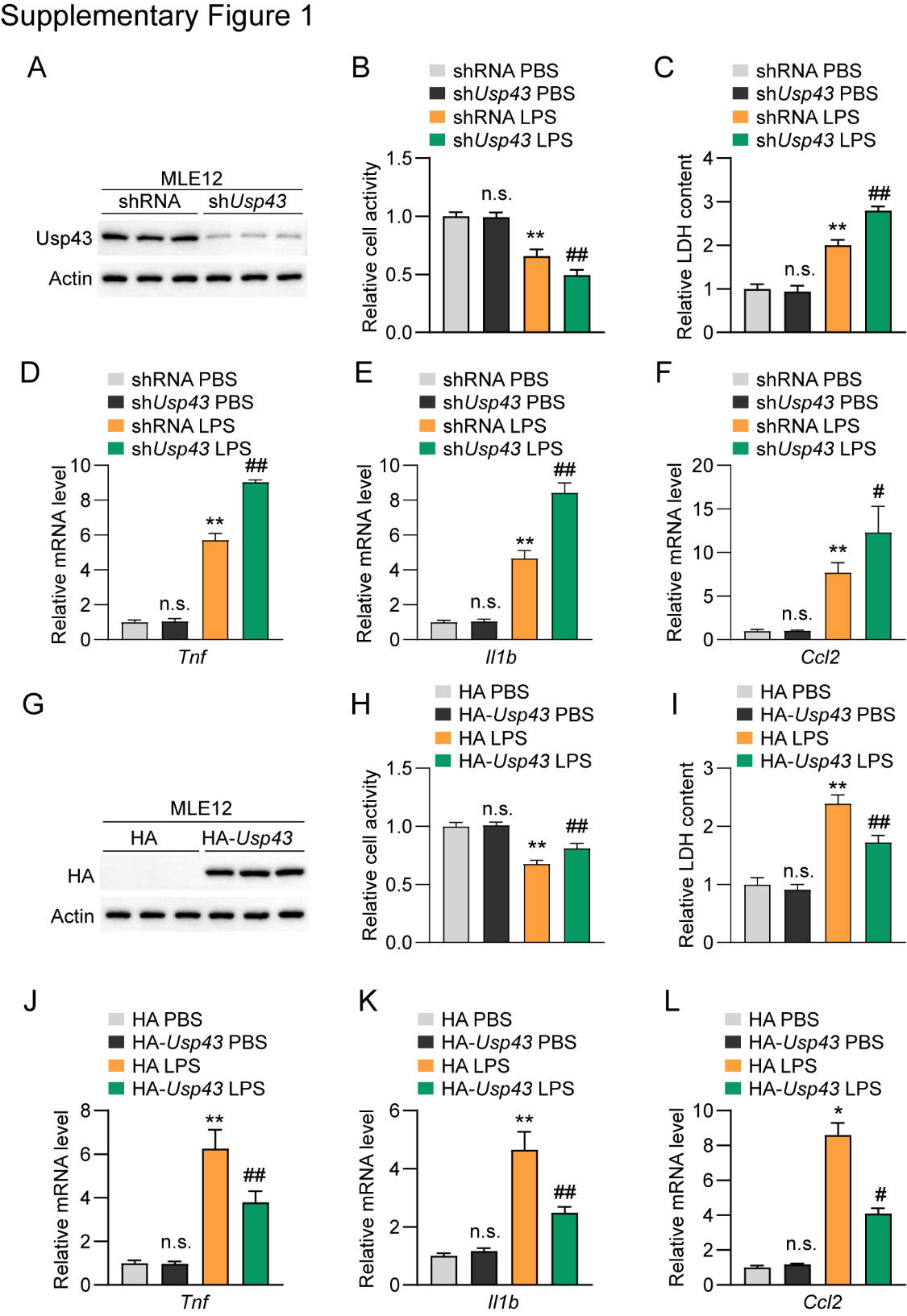


**Supplementary Figure 1. USP43 modulates LPS-induced cell injury and inflammatory responses in MLE12 cells.** (A) Western blot detection results of Usp43 in Usp43-knockdown (sh*Usp43*) and control (shRNA) MLE12 cells. (B) The CCK8 assay results for cell viability of sh*Usp43* and shRNA cells treated with PBS or LPS. (C) Relative LDH content detection results of sh*Usp43* and shRNA cells treated with PBS or LPS. (D-F) Relative mRNA expression of inflammatory cytokines (*Tnf* (D)*, Il1b* (E)*, and Ccl2* (F)) in sh*Usp43* and shRNA cells after PBS or LPS treatment. (G) Western blot detection results of Usp43 in Usp43-overexpression (HA-*Usp43*) and control (HA) MLE12 cells. (H) The CCK8 assay results for cell viability of HA-*Usp43* and control cells treated with PBS or LPS. (I) Relative LDH content detection results of HA-*Usp43* and control cells treated with PBS or LPS. (J-L) Relative mRNA expression of inflammatory cytokines (*Tnf, Il1b, and Ccl2*) in HA-USP43 and control cells after PBS or LPS treatment. n= 3 independent biological repetitions. The one-way ANOVA test followed by Bonferroni’s post hoc test or Tamhane’s T2 (M) post hoc test was used for statistical analysis. n.s., no significance vs shRNA PBS group or HA PBS group. *, P<0.05, ** P<0.01 vs shRNA PBS group or HA PBS group. #, P<0.05, ## P<0.01 vs shRNA LPS group or HA LPS group.


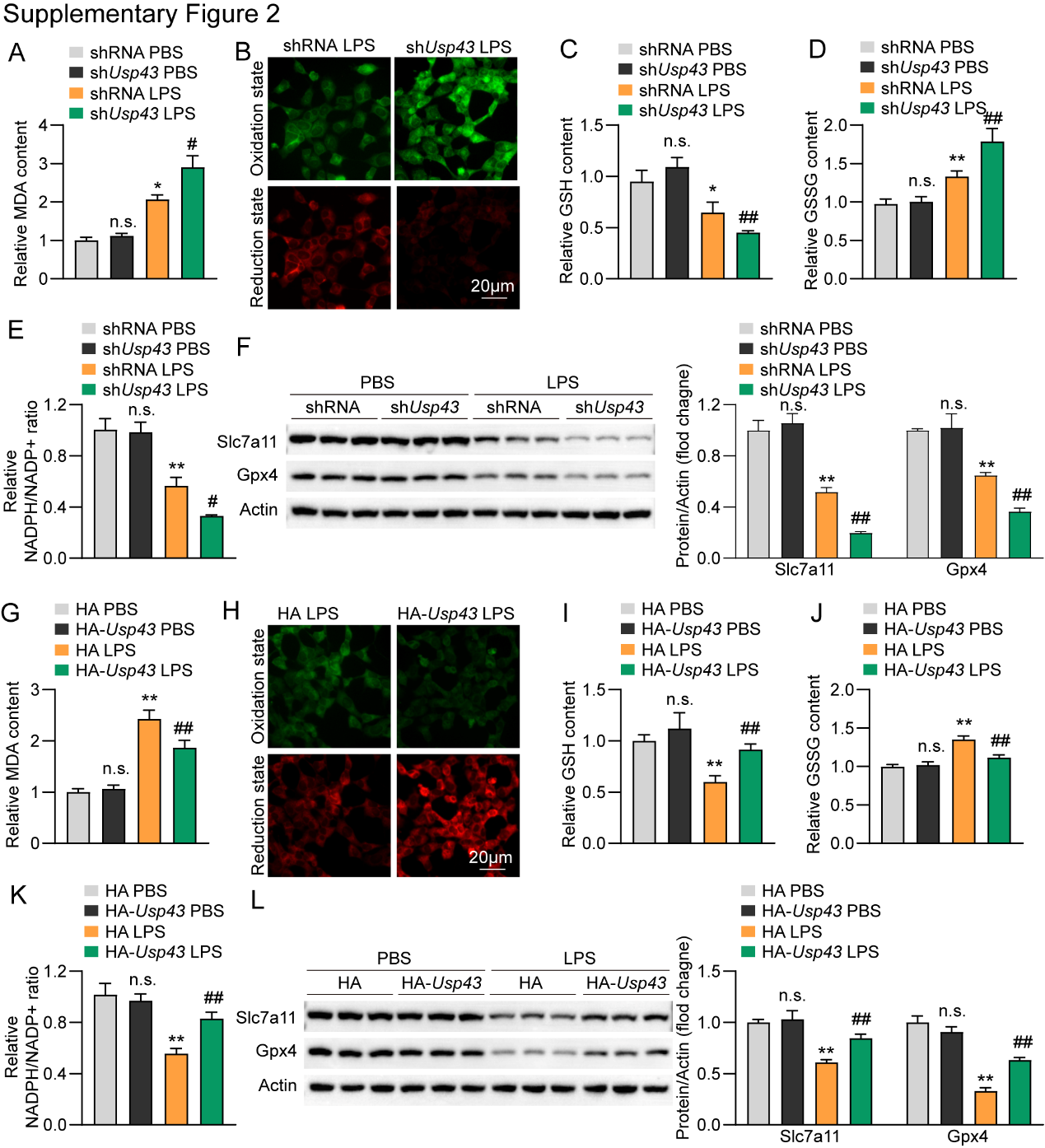


**Supplementary Figure 2. Usp43 knockdown exacerbates while its overexpression alleviates LPS-induced ferroptosis in MLE12 cells.** (A) Relative MDA contents in Usp43-knockdown and control MLE12 cells after PBS or LPS treatment (n= 4 independent biological repetitions). (B) Representative immunofluorescence image of lipid peroxidation staining using the BODIPY 581/591 C11 kit (n= 3 independent biological repetitions). (C-E) Relative GSH contents (C), GSSG contents (D) and NADPH/NADP^+^ ratio (E) in Usp43-knockdown and control MLE12 cells after PBS or LPS treatment (n= 4 independent biological repetitions). (F) Western blot analysis (Left) and quantitative result (Right) of Slc7a11 and Gpx4 protein levels in cells with the indicated treatment (n= 3 independent biological repetitions). (G) Relative MDA contents in Usp43-overexpression and control cells after PBS or LPS treatment (n= 4 independent biological repetitions). (H) Representative immunofluorescence image of lipid peroxidation staining using the BODIPY 581/591 C11 kit (n= 3 independent biological repetitions). (I-K) Relative GSH contents (I), GSSG contents (J) and NADPH/NADP^+^ ratio (K) in Usp43- overexpression and control cells after PBS or LPS treatment (n= 4 independent biological repetitions). (L) Western blot analysis (Left) and quantitative result (Right) of Slc7a11 and Gpx4 protein levels in cells with the indicated treatment (n= 3 independent biological repetitions). The one-way ANOVA test followed by Bonferroni’s post hoc test or Tamhane’s T2 (M) post hoc test was used for statistical analysis. n.s., no significance vs shRNA PBS group or HA PBS group. *, P<0.05, ** P<0.01 vs shRNA PBS group or HA PBS group. #, P<0.05, ## P<0.01 vs shRNA LPS group or HA LPS group.


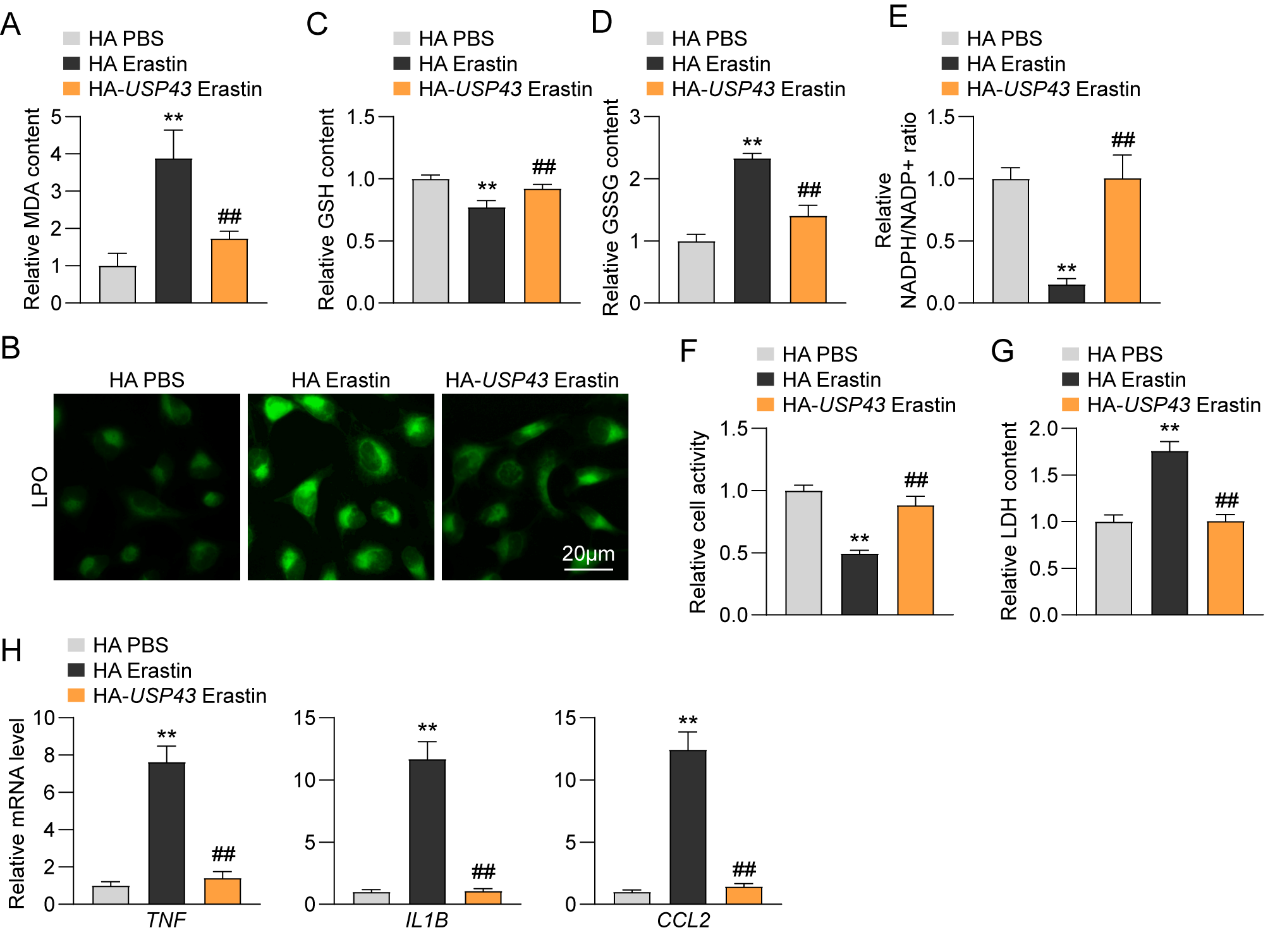


**Supplementary Figure 3. USP43 alleviates Erastin-induced ferroptosis in BEAS-2B cells.** (A) Relative MDA contents in USP43 overexpressed BEAS-2B cells and control cells that treat with Erastin or PBS (n= 4 independent biological repetition). (B) Representative immunofluorescence staining image of lipid peroxidation using the BODIPY 581/591 C11 kit in the indicated groups (n= 3 independent biological repetition). (C-E) Relative GSH contents (C), GSSG contents (D) and NADPH/NADP^+^ ratio (E) in USP43 overexpressed BEAS-2B cells and control cells that treat with Erastin or PBS (n= 4 independent biological repetition). (F) Relative cell activity assessed result by CCK8 assay in the indicated groups (n= 3 independent biological repetition). (G) Relative LDH content analysis result in the medium from the indicated groups (n= 3 independent biological repetition). (H) Relative mRNA expression of inflammatory cytokines (*TNF, IL1B, and CCL2*) in cells from the indicated groups (n= 4 independent biological repetition). The one-way ANOVA analysis followed by Bonferroni’s post hoc test or Tamhane’s T2 (M) post hoc test was used for statistical analysis. *, P<0.05, ** P<0.01 vs HA PBS group. #, P<0.05, ## P<0.01 vs HA Erastin group.


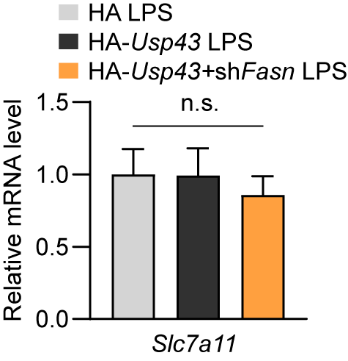


**Supplementary Figure 4. The regulation of Slc7a11 by Usp43 is not dependent on Fasn in LPS-induced MLE12 cells.**


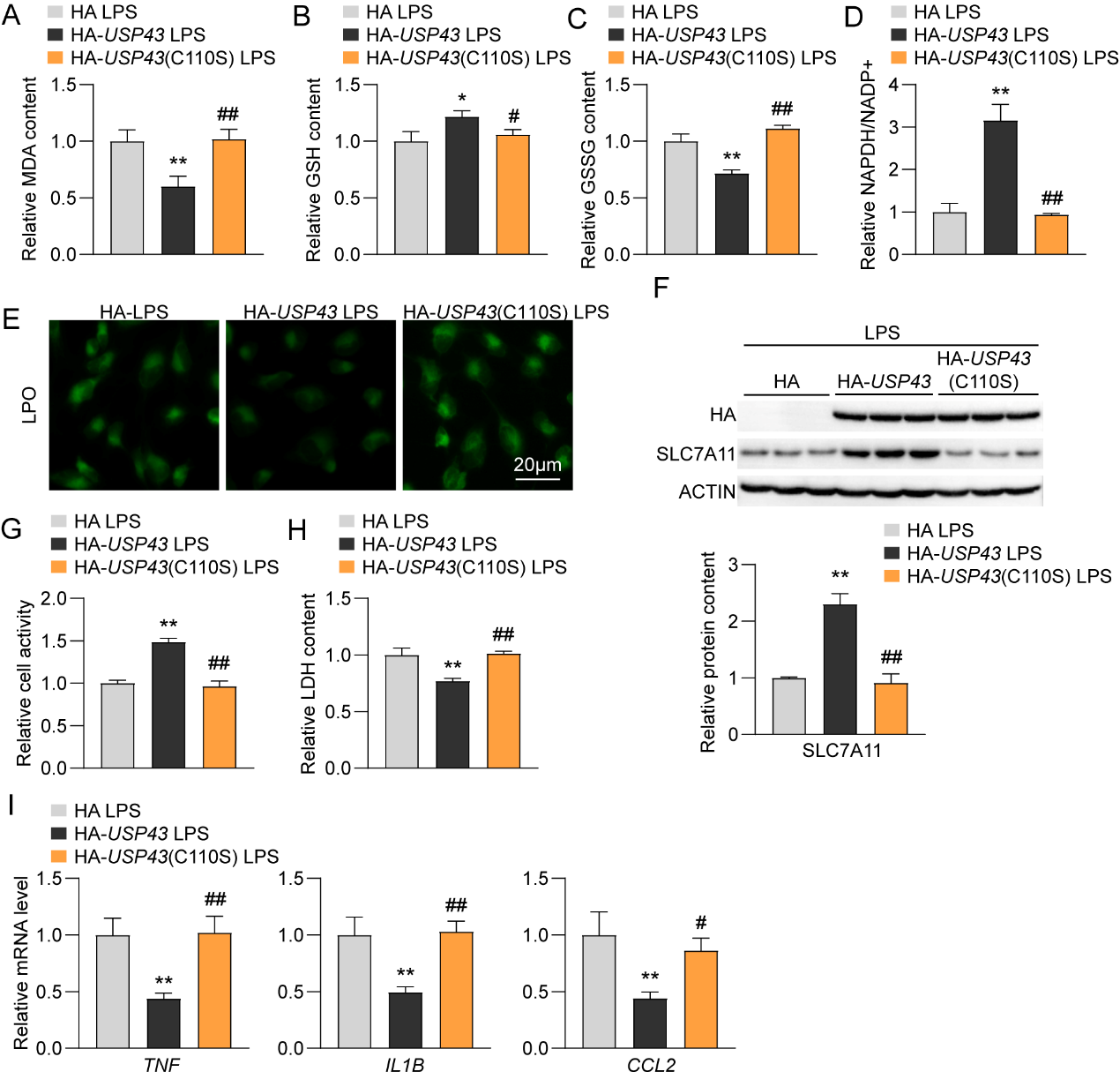


**Supplementary Figure 5. USP43 activity mutation abolished its protective effect against LPS-induced ferroptosis, cell damage, and inflammatory response.** (A) Relative MDA contents in USP43 or USP43(C110S) overexpressed BEAS-2B cells and control cells that treat with LPS (n= 4 independent biological repetition). (B) Representative immunofluorescence staining image of lipid peroxidation using the BODIPY 581/591 C11 kit in the indicated groups (n= 3 independent biological repetition). (C-E) Relative GSH contents (C), GSSG contents (D) and NADPH/NADP^+^ ratio (E) in cells from the indicated groups (n= 4 independent biological repetition). (F) Western blot analysis of SLC7A11 and HA-USP43 expression in BEAS-2B cells from the indicated groups (n= 3 independent biological repetition). (G) Relative cell activity assessed result by CCK8 assay in the indicated groups (n= 3 independent biological repetition). (H) Relative LDH content analysis result in the medium from the indicated groups (n= 3 independent biological repetition). (I) Relative mRNA expression of inflammatory cytokines (*TNF, IL1B,* and *CCL2*) in cells from the indicated groups (n= 4 independent biological repetition). The one-way ANOVA analysis followed by Bonferroni’s post hoc test or Tamhane’s T2 (M) post hoc test was used for statistical analysis. *, P<0.05, ** P<0.01 vs HA LPS group. #, P<0.05, ## P<0.01 vs HA-*USP43* LPS group.


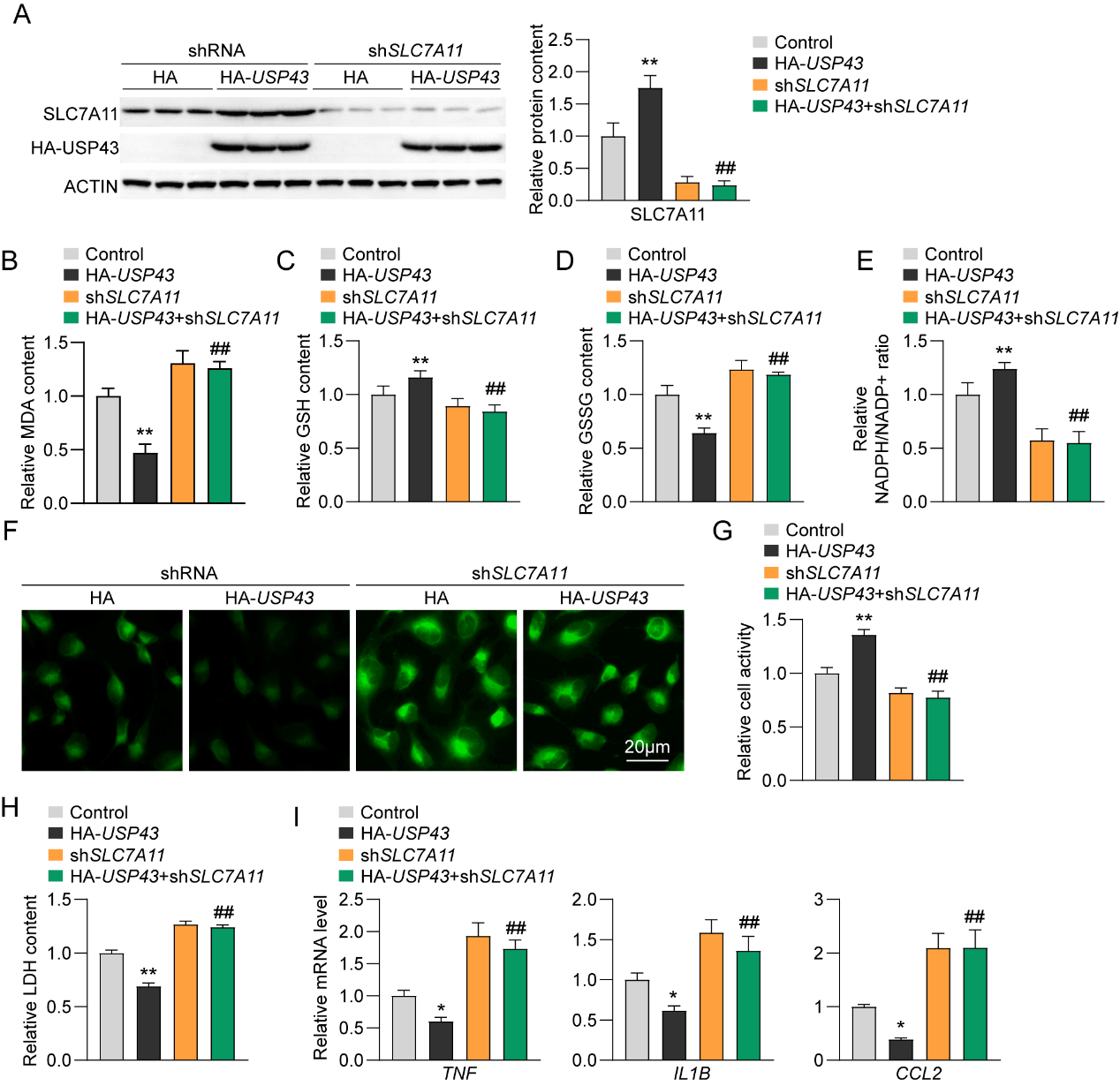


**Supplementary Figure 6. Knockdown of SLC7A11 rescued the protecting effect of USP43 overexpression on ALI.** (A) Western blot analysis of SLC7A11 and HA-USP43 expression in BEAS-2B cells with USP43 overexpression and/or SLC7A11 knockdown (n= 3 independent biological repetition). (B) Relative MDA contents in LPS-treated BEAS-2B cells with USP43 overexpression and/or SLC7A11 knockdown (n= 4 independent biological repetition). (C) Representative immunofluorescence image of lipid peroxidation staining using the BODIPY 581/591 C11 kit in the indicated groups (n= 3 independent biological repetition). (D-F) Relative GSH contents (D), GSSG contents (E) and NADPH/NADP^+^ ratio (F) in LPS-treated BEAS-2B cells with USP43 overexpression and/or SLC7A11 knockdown (n= 4 independent biological repetition). (G) Relative cell activity assessed result by CCK8 assay in the indicated groups (n= 3 independent biological repetition). (H) Relative LDH content analysis result in the medium from the indicated groups (n= 3 independent biological repetition). (I) Relative mRNA expression of inflammatory cytokines (*TNF, IL1B, and CCL2*) in cells from the indicated groups (n= 4 independent biological repetition). (J) Elisa quantification of secreted inflammatory cytokines (TNF-α, IL-1β, CCL2) in cell culture supernatants from the indicated groups (n= 4 independent biological repetition). The one-way ANOVA analysis followed by Bonferroni’s post hoc test or Tamhane’s T2 (M) post hoc test was used for statistical analysis. *, P<0.05, ** P<0.01 vs Control LPS group. #, P<0.05, ## P<0.01 vs HA-USP43 LPS group.
