# Supplementary material for: USP43‐mediated deubiquitination of SLC7A11 protects against LPS‐induced acute lung injury by inhibiting ferroptosis
Source: Clin Transl Med. 2026 Jun 30;16(7):e70718. doi: 10.1002/ctm2.70718 (PMC13319409; doi:10.1002/ctm2.70718)
Supplement: Supplementary file 2 — Supporting Information [file CTM2-16-e70718-s001.docx]

Supplementary Table 1. The primer sequences used for USP43 and SLC7A11 overexpression and knockdown

| Primer | Species | Sequences 5’-3’ | |
| --- | --- | --- | --- |
| HA-USP43 | Human | F | TCGGGTTTAAACGGATCCATGGACCTGGGCCCCG |
|  |  | R | GGGCCCTCTAGACTCGAGTCAAAAGCTGGACTCCGGTAAGG |
| sh*USP43* | Human | F | CCGGGGAGTTCAAGAATGCAGTTTCCTCGAGGAAACTGCATTCTTGAACTCCTTTTTG |
|  |  | R | AATTCAAAAAGGAGTTCAAGAATGCAGTTTCCTCGAGGAAACTGCATTCTTGAACTCC |
| Flag-SLC7A11 | Human | F | TCGGGTTTAAACGGATCCATGGTCAGAAAGCCTGTTGTGT |
|  |  | R | GGGCCCTCTAGACTCGAGTCATAACTTATCTTCTTCTGGTACAACTTCCAGT |
| sh*SLC7A11* | Human | F | CCGGCCTGTCACTATTTGGAGCTTTCTCGAGAAAGCTCCAAATAGTGACAGGTTTTTG |
|  |  | R | AATTCAAAAACCTGTCACTATTTGGAGCTTTCTCGAGAAAGCTCCAAATAGTGACAGG |
| HA-Usp43 | Mouse | F | ACGCGTGGTCTCGGATCCGCCACCATGGATCCGGGCGTGGGG |
|  |  | R | TCTAGACGTCTCCTCGAGGAAGCTGGACTCAGGTAAGGATTTCTTTCGAC |
| sh*Usp43* | Mouse | F | CCGGGAAGATGGTTGVAGAGGAAGGCTCGAGCCTTCCTCTGCAACCATCTTCTTTTTG |
|  |  | R | AATTCAAAAAGAAGATGGTTGCAGAGGAAGGCTCGAGCCTTCCTCTGCAACCATCTTC |

Supplementary Table 2. The primer sequences used for RT-PCR.

| Gene | Primer | Sequences |
| --- | --- | --- |
| Human *ACTB* | Forward | 5’- CTCGCCTTTGCCGATCC-3’ |
|  | Reverse | 5’- TTCTCCATGTCGTCCCAGTTG -3’ |
| Human *TNF* | Forward | 5’- TATCCTGGGGGACCCAATGT -3’ |
|  | Reverse | 5’- AAAGAAGGCACAGAGGCCAG -3’ |
| Human *IL1B* | Forward | 5’- CAGAAGTACCTGAGCTCGCC -3’ |
|  | Reverse | 5’- AGATTCGTAGCTGGATGCCG -3’ |
| Human *CCL2* | Forward | 5’- CGCCTCCAGCATGAAAGTCT -3’ |
|  | Reverse | 5’- GAGCCCTTGGGGAATGAAGG -3’ |
| Human *USP43* | Forward | 5’- CAAGGTGGGCATTACACAGC -3’ |
|  | Reverse | 5’- CCTCATCTTCTCGAAGCGGT -3’ |
| Mouse *Actb* | Forward | 5’- TGAGCTGCGTTTTACACCCT-3’ |
|  | Reverse | 5’- GCCTTCACCGTTCCAGTTTT -3’ |
| Mouse *Tnf* | Forward | 5’- ATGGCCTCCCTCTCATCAGT -3’ |
|  | Reverse | 5’- TTTGCTACGACGTGGGCTAC -3’ |
| Mouse *Il1b* | Forward | 5’- GCCACCTTTTGACAGTGATGAG -3’ |
|  | Reverse | 5’- TGATGTGCTGCTGCGAGATT -3’ |
| Mouse *Il6* | Forward | 5’- CCCCAATTTCCAATGCTCTCCT -3’ |
|  | Reverse | 5’- CATAACGCACTAGGTTTGCCG -3’ |
| Mouse *Ccl2* | Forward | 5’- TGCCCTAAGGTCTTCAGCAC -3’ |
|  | Reverse | 5’- AAGGCATCACAGTCCGAGTC -3’ |
| Mouse *Cxcl10* | Forward | 5’- GGTCTGAGTCCTCGCTCAAG -3’ |
|  | Reverse | 5’- GTCGCACCTCCACATAGCTT-3’ |
| Mouse *Usp43* | Forward | 5’- CGCTGGACTTCCTCTACGAC -3’ |
|  | Reverse | 5’- CAGGGAGTTCCGGCAATAGG -3’ |

Supplementary Table 3. The primers used for plasmid construction.

| Primer | Sequences 5’-3’ | |
| --- | --- | --- |
| Flag-SLC7A11 | F | TCGGGTTTAAACGGATCCATGGTCAGAAAGCCTGTTGTGT |
|  | R | GGGCCCTCTAGACTCGAGTCATAACTTATCTTCTTCTGGTACAACTTCCAGT |
| Flag-GPX4 | F | TCGGGTTTAAACGGATCCATGAGCCTCGGCCGCCT |
|  | R | GGGCCCTCTAGACTCGAGGTGCACGCTGGATTTTCGGG |
| HA-USP43 | F | TCGGGTTTAAACGGATCCATGGACCTGGGCCCCG |
|  | R | GGGCCCTCTAGACTCGAGTCAAAAGCTGGACTCCGGTAAGG |
| GST-HA-USP43 | F | TCGGGTTTAAACGGATCCATGGACCTGGGCCCCG |
|  | R | GGGCCCTCTAGACTCGAGTCAAAAGCTGGACTCCGGTAAGG |
| GST-HA-SLC7A11 | F | TCGGGTTTAAACGGATCCATGGTCAGAAAGCCTGTTGTGT |
|  | R | GGGCCCTCTAGACTCGAGTCATAACTTATCTTCTTCTGGTACAACTTCCAGT |
| Flag-USP43 | F | TCGGGTTTAAACGGATCCATGGACCTGGGCCCCG |
|  | R | GGGCCCTCTAGACTCGAGTCAAAAGCTGGACTCCGGTAAGG |
| Flag-USP43(1-712) | F | TCGGGTTTAAACGGATCCATGGACCTGGGCCCCG |
|  | R | GGGCCCTCTAGACTCGAGTCAGCTGTTCCGCTTCTGATAGAAC |
| Flag-USP43(713-1123) | F | TCGGGTTTAAACGGATCCATCCCTCCCTGGTCAGCCAG |
|  | R | GGGCCCTCTAGACTCGAGTCAAAAGCTGGACTCCGGTAAGG |
| HA-SLC7A11 | F | TCGGGTTTAAACGGATCCATGGTCAGAAAGCCTGTTGTGT |
|  | R | GGGCCCTCTAGACTCGAGTCATAACTTATCTTCTTCTGGTACAACTTCCAGT |
| HA-SLC7A11(44-501) | F | TCGGGTTTAAACGGATCCGTCACTTTACTGAGGGGAGTCTC |
|  | R | GGGCCCTCTAGACTCGAGTCATAACTTATCTTCTTCTGGTACAACTTCCAGT |
| HA-SLC7A11(1-470) | F | TCGGGTTTAAACGGATCCATGGTCAGAAAGCCTGTTGTGT |
|  | R | GGGCCCTCTAGACTCGAGTCACCATATAATAAAGAGATAATACGCAGGGAC |
| HA-USP43-C110S | F | CACGGCAACACCTCTTTCATGAACGC |
|  | R | GCGTTCATGAAAGAGGTGTTGCCGTG |
